# Dual-Piezo-Charge Strategy in (1–0)–3 Single-Crystal Composite for Enhancing Underwater Acoustic Sensing

**DOI:** 10.1007/s40820-026-02102-1

**Published:** 2026-02-28

**Authors:** Yu Lei, Xiaotian Li, Zewei Hou, Bing Wang, Shuguang Zheng, Yang Wei, Zhonghui Yu, Jiawang Hong, Shuxiang Dong

**Affiliations:** 1 School of Aerospace Engineering, Beijing Institute of Technology, Beijing, 100081 People’s Republic of China; 2 School of Materials Science and Engineering, Peking University, Beijing, 100871 People’s Republic of China; 3 College of Semiconductors (College of Integrated Circuits), Hunan University, Changsha, 410082 People’s Republic of China; 4 Beijing Institute of Technology, Zhuhai, 519088 People’s Republic of China; 5 Institute for Advanced Study, Shenzhen University, Shenzhen, 518061 People’s Republic of China; 6 Electronic Materials Research Laboratory, Key Laboratory of the Ministry of Education and International Center for Dielectric Research, School of Electronic Science and Engineering, Xi’an Jiaotong University, Xi’an, 710049 People’s Republic of China; 7 College of Mechanical and Electrical Engineering, Hohai University, Changzhou, 213200 People’s Republic of China; 8 State Key Laboratory of Environment Characteristics and Effects for Near-Space, Beijing Institute of Technology, Beijing, 100081 People’s Republic of China

**Keywords:** (1–0)–3 single crystal piezocomposite, Dual-piezo-charge mechanism, Hydrostatic figure of merit, Piezoelectric ultrasonic transducers, Acoustic sensitivity

## Abstract

Piezoelectric ultrasonic transducers (PUTs) are extensively used in diverse technological fields, yet further enhancement in acoustic sensitivity and hydrostatic figure of merit* d*_h_*g*_h_ have plateaued in conventional 1–3 piezocomposites, whose structures have remained largely unchanged for decades. Herein, we report a (1–0)–3 single-crystal piezocomposite (SCPC) based on the [011]-oriented Pb(In_1/2_Nb_1/2_)O_3_-Pb(Mg_1/3_Nb_2/3_)O_3_-PbTiO_3_ (PIN-PMN-PT) single-crystal, featuring a dual-piezo-charge (DPC) mechanism arising from the piezoelectric and piezoelectret synergy effect. The ordered and polar 0-phase microholes with aligned dipoles are intentionally introduced in 1-phase piezo-pillars, yielding a uniquely low effective dielectric constant, reduced acoustic impedance, and DPC properties unattainable in conventional SCPCs. Both Simulations and experiments confirm that this DPC mechanism in (1–0)–3 SCPC enables an ultrahigh *d*_h_*g*_h_ of ~ 8089 × 10^−15^ m^2^ N^−1^, representing increases of 443.2% and 890.6% over commercial 1–3 SCPC and 1–3 piezoceramic composite, respectively. The engineered microhole structure and DPC strategy also significantly enhance underwater acoustic sensitivity while maintaining a broad − 3 dB bandwidth of 130 kHz, crucial for maritime safety and collision avoidance. Meanwhile, the long-term testing further verifies the PUT’s operational stability. This design approach offers a promising pathway for next-generation piezocomposites and PUT technologies.

**Supplementary Information:**

The online version contains supplementary material available at 10.1007/s40820-026-02102-1.

## Highlights

- (1–0)–3 single-crystal piezocomposite (SCPC) based on [011]-oriented relaxor PIN-PMN-PT crystal was fabricated via a modified method combining 3D printing-assisted dice-and-insert with dice-and-fill techniques.
- The (1–0)–3 SCPC, characterized by ordered and poled microholes and incorporating a dual-piezo-charge mechanism, achieves an ultrahigh hydrostatic figure of merit *d*_h_*g*_h_ of about 8089 × 10^−15^ m^2^ N^−1^.
- The ultrasound transducer based on (1–0)–3 SCPC exhibits a ~ 70% enhancement in underwater acoustic sensitivity while maintaining a broad − 3 dB bandwidth of 130 kHz.

**Supplementary Information:**

The online version contains supplementary material available at 10.1007/s40820-026-02102-1.

## Graphical abstract

**Figure:**
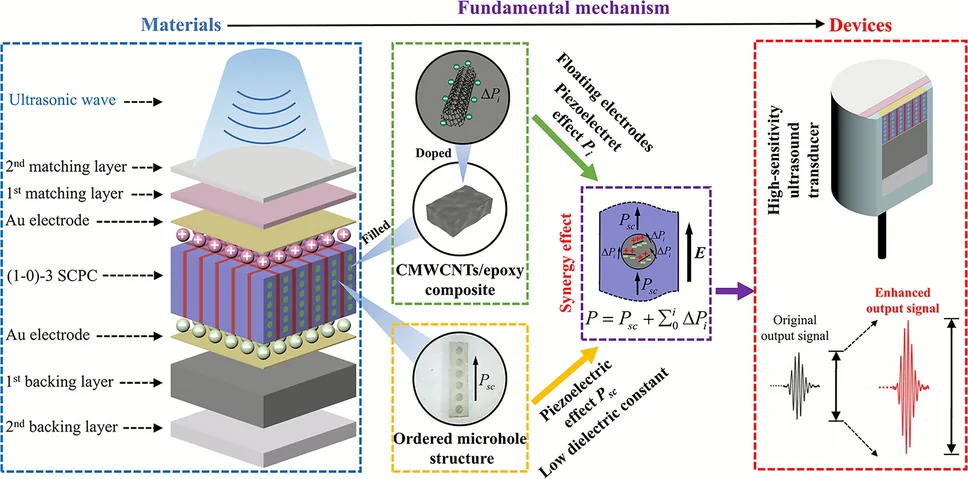

## Introduction

Ultrasonic technology has been widely adopted across various technological and biomedical fields owing to its inherent advantages, including safety, non invasiveness, real-time imaging capability, and strong penetration depth. Among the diverse ultrasonic devices, piezoelectric ultrasonic transducers (PUTs) have gained particular prominence and are extensively utilized in applications such as human tissue volumetric imaging [1], continuous and non invasive monitoring [2, 3], noninvasive therapy [4], energy harvesting [5], underwater detection and navigation [6], ultrasonic catalysis [7] and non-destructive testing [8]. In the past decades, various types of piezoelectric materials have undergone rapid development. The perovskite lead zirconate titanate (PZT) ceramics have been widely used in ultrasonic transducers due to their great commercial success [9, 10]. With continuous scientific advancements, lead-free ceramics incorporating defect engineering [11, 12], textured ceramics [13, 14] and relaxor ferroelectric single crystal [15, 16] with superior piezoelectric properties have emerged. The ultrahigh piezoelectric charge coefficients *d*_33_ of these materials can significantly enhance the transmission performance of transducer, while their piezoelectric voltage coefficients *g*_33_ are still low due to ultrahigh dielectric permittivity, which is not conducive to the receiving sensitivity of the transducers. Meanwhile, polyvinylidene fluoride (PVDF) ferroelectric polymers [17, 18] and piezoelectric textiles [19, 20] exhibit nearly opposite physical and piezoelectric properties compared to ceramics and single crystals, resulting in higher receiving sensitivity and flexibility for transducers, but weaker transmitting performance.

To overcome the limitations of monolithic material and enhance overall practicality and compatibility, piezoelectric composites, which combine piezoelectric phases with non-piezoelectric matrices, have undergone rapid development, providing enhanced flexibility, tunable properties, and improved electromechanical performance for diverse applications [21]. Among the different connectivity types of piezoelectric composites, 1–3 type composites have attracted extensive research interest due to their high electromechanical conversion efficiency, mechanical stability, sensitivity, low acoustic impedance, and moderate flexibility [22–27]. However, despite their performance advantages, 1–3 piezoelectric composites still face bottlenecks arising from the intrinsic limitations of their constituent materials. In recent years, relaxor piezoelectric single-crystal with excellent piezoelectric coefficients and electromechanical coupling factors employed to replace the piezoceramics in composites has shown great potential for enhancing transducer performance [28–33]. However, if the modification is restricted to the piezoelectric material, the performance typically reaches an upper limit determined by its volume fraction [34]. Therefore, the composite structure, which plays a decisive role in the overall performance of PUTs, is equally critical [35, 36].

Normally, substituting a portion of the epoxy resin in the composite with softer silicone rubber can alleviate the clamping effect of the passive phase on the piezoelectric pillars, thereby enhancing the electromechanical coupling coefficient [26]. Furthermore, the propagation of spurious mode vibration can be cut off by soft polymer when the rubber content exceeds that of the epoxy [37]. However, the enhancement achieved by using polymers with varying stiffness is marginal, whereas composite materials with more intricate structures show more substantial performance improvements. A novel (1_A_, 1_B_)–3 piezocomposite structure containing two types of single-crystal pillars, and the combination effect and synergistic action of two different piezo-pillars notably broaden the working bandwidth and improve the sound sensitivity [24]. A non-periodic 1–3 piezocomposites structure, fabricated by synchronously varying the widths of PZT-5H ceramic and epoxy resin in opposite trend, can suppress or weaken the impact of transverse vibration modes, thereby improving the sensitivity of the ultrasonic transducer [38]. Moreover, piezocomposites with other complex structures such as multilayer stairstep type [39], super-cell type [40], and Gaussian type [41] have also been designed to reduce the effect of lateral redundant modes and broaden the bandwidth.

On the other hand, introducing porosity in piezoelectric materials has demonstrated intriguing potential for enhancing the performance of piezoelectric devices [42–47]. Generally, the benefit of incorporating porosity is to achieve a fast reduction in dielectric constant, thereby increasing the sensitivity of piezoelectric sensors since their figures of merit (FOMs) are inversely proportional to this decreasing value. A multilevel structural engineering that combines sandwich porous (dense/porous/dense) and 1–3 ceramic composite structures is exploited to enhance both *g*_33_ (~ 61.4 × 10^–3^ V m N^−1^) and *d*_33_*g*_33_ (17,806 × 10^–15^ m^2^ N^−1^) values remarkably, even superior to some textured lead-based ceramics [48]. The porous PZT ceramics with a well-designed ordered pore structure, fabricated using freeze-casting and 3D printing techniques, can simultaneously offer high piezoelectric constants and ultra-low dielectric constants [49–52]. However, due to insufficient polarization, the porous structure may result in decreased piezoelectric constants, particularly at higher porosity levels [53]. For example, the *d*_33_ of 3–3-type porous piezoelectric materials decreases from 470 to 270 pC N^−1^ after introducing 20.7 vol% porosity [46]. This trade-off can even offset the advantages of introducing porous structures, as the benefits of reduced dielectric constants may be negated by the degradation of piezoelectric properties, thereby limiting the full potential of porous piezoelectric materials [44, 45]. In addition, although numerous researches have demonstrated that porous structure can significantly enhance the receiving performance of piezoelectric ceramic transducers [48–50, 54], the feasibility of this design strategy in piezoelectric single-crystal systems with greater development potential remains to be validated due to manufacturing technology limitations.

In this work, the [011]-oriented relaxor ferroelectric Pb(In_1/2_Nb_1/2_)O_3_-Pb(Mg_1/3_Nb_2/3_)O_3_-PbTiO_3_ (PIN-PMN-PT) single crystal is used as piezoelectric phase, while in terms of composite structure, for the first time, we presented a novel (1–0)–3 single-crystal piezocomposite (SCPC) featuring the piezo-pillars (terming as 1-phase) that incorporate ordered and poled microhole structure (serving as 0-phase), meticulously fabricated through precision machining. Meanwhile, the epoxy resin doped with 0.25 wt% carboxylated multi-walled carbon nanotubes (CMWCNTs) is filled into microholes of the crystal pillars, enabling 0-phase microholes to effectively mitigate the adverse effects of porosity on the polarization response of the single crystal and provide piezoelectret effect under the influence of a strong electric field. The microhole structure within the SCPC is adequately utilized to reduce the dielectric constant; meanwhile, the piezoelectret effect induced by poled microholes, combined with the piezoelectric effect of the piezo-pillars, gives rise to a dual-piezo-charge (DPC) mechanism. The DPC mechanism can further enhance the piezoelectric response to incident acoustic waves. Thereby, the hydrostatic figure of merit *d*_h_*g*_h_ of the SCPC is improved, achieving a value of 8088.9 × 10^–15^ m^2^ N^−1^. Moreover, passive phase (epoxy resin terming as 3-phase) widths along the horizontal direction in piezocomposite were designed to be inconsistent, which can eliminate and weaken the effect of lateral modes. Consequently, two kinds of PUTs based on novel (1–0)–3 SCPC containing ordered and polar 0-phase microholes and conventional 1–3 SCPC were fabricated. Compared with the PUT based on conventional piezocomposite, the PUT based on the (1–0)–3 SCPC shows superior performance, including a better receiving voltage sensitivity of − 184.3 dB, a − 3dB receiving frequency bandwidth of 130 kHz, as well as a 68.1% increase in maximum output voltage during the pulse-echo experiment. The long-term stability and fatigue resistance performances of the SCPC-based transducer have also been demonstrated through prolonged operation. The proposed strategy offers valuable insights for further enhancing the performance of 1–3 piezocomposite materials and points to a promising direction for the development of next-generation ultrasonic transducers.

## Experimental Section

### Materials

All single crystal was supplied by Suzhou Institute of Electronic Functional Materials Technology Co., Ltd. (China). The epoxy resin (EPO-TEK 301) and the conductive silver epoxy (EPO-TEK H20E) were purchased from Epoxy Technology Co., Ltd. (USA). The carboxylated multi-walled carbon nanotubes were purchased from Shenzhen Suiheng Technology Co., Ltd. (China). The zirconia oxide powder (5 µm, 99%) was purchased from Sigma-Aldrich Inc. (USA). The tungsten (W) powder (APS 1–5 µm, 99.9%) was purchased from Alfa Aesar Co., Ltd. (USA). The hollow glass microspheres (S32, 30–50 µm) were purchased from 3M Co., Ltd. (USA).

### Properties Characterization of SCPCs

The piezoelectric charge coefficients *d*_3j_ (j = 1, 2, 3) were directly measured by the quasi-static *d*_33_ meter (BLBZJ6AN, Balab Technologies). The capacitance of the piezocomposites was measured using an impedance analyzer (E4990A, Keysight Technologies) at a frequency of 1 kHz. The remaining material parameters were calculated indirectly using the formulas provided in Text S1.

### Properties Characterization of PUTs

#### Receiving Voltage Sensitivity

Within the frequency range of 300–600 kHz, the input voltage frequency was swept in 10 kHz increments, and the corresponding output voltage was recorded using a calibrated hydrophone. The sound pressure generated by the calibrated transmitting transducer was determined based on the output voltage amplitude measured by the hydrophone. Subsequently, the calibrated hydrophone was replaced with the fabricated transducer to evaluate its receiving performance. The same measurement procedure was repeated, and the output voltage signal from the fabricated transducer was recorded. The receiving voltage sensitivity was then calculated using the following formula:

1 $$RVS = 20\log \frac{{V_{out} /P_{out} }}{1V/uPa}$$

where $P_{out}$ is the sound pressure perceived by the calibrated transmitting transducer and $V_{out}$ is the output voltage signal generated by the fabricated transducer.

#### Self-Transmit-Receive Performance

The self-transmit-receive performance of the transducers was evaluated through pulse-echo experiments conducted in an ultrapure water tank. The radiating surface of the transducer was aligned parallel to the quartz plate used as the reflector, with a fixed spacing of 50 mm between them. A sinusoidal pulse signal with 3 cycles and a peak-to-peak value amplitude of 5 V were used as the input voltage. The trigger interval of the pulse signal was set to be 1 ms. Similarly, the input voltage frequency was swept in 10 kHz increments, while the corresponding output voltage signal was recorded in real time. The frequency spectrum of transducer was obtained from the echo signal and its Fourier transform.

#### Fatigue Resistance

A sinusoidal pulse signal with 10 cycles and a peak-to-peak amplitude of 5 V were used as the input signal to enable clear observation of changes in the output signal. Meanwhile, the trigger interval of the pulse signal was set to be 100 μs to ensure that the transducer remains in the active state as much as possible without compromising the readability of the output signal.

## Results and Discussion

### Fabrication and Characterization of (1–0)–3 SCPC

The electromechanical coupling performance of piezoelectric composites directly affects the overall performance of transducers. For clarity, the critical parameters of the piezocomposites are provided in Text S1. Moreover, Text S2 presents a detailed discussion on how the microhole structure affects the performance of SCPCs, along with its underlying mechanisms. Based on the series and parallel theoretical models of piezoelectric composites, aligning the pores parallel to the poling direction on the single-crystal pillars can minimize the adverse effects of microholes on the polarization response. Previous research findings also support the rationality of this type of pore arrangement [45, 50]. Ultimately, considering the trade-off between microholes fabrication difficulty and performance enhancement, a novel structure with 6 microholes and hole diameter of 0.4 mm is selected in this work. However, although the ordered microhole structure is beneficial to the overall polarization response, the adverse effect cannot be eliminated completely. Therefore, an appropriate amount of CMWCNTs is incorporated into the epoxy resin to address this issue in this work. It is worth noting that the CMWCNTs must be uniformly dispersed within the epoxy matrix, and their mass ratio should not exceed 0.25% to prevent the formation of conductive pathways in the composites. Further details regarding the effect of CMWCNTs on the composite properties and the CMWCNTs/epoxy composites preparation process are provided in Text S3.

**Fig. 1:**
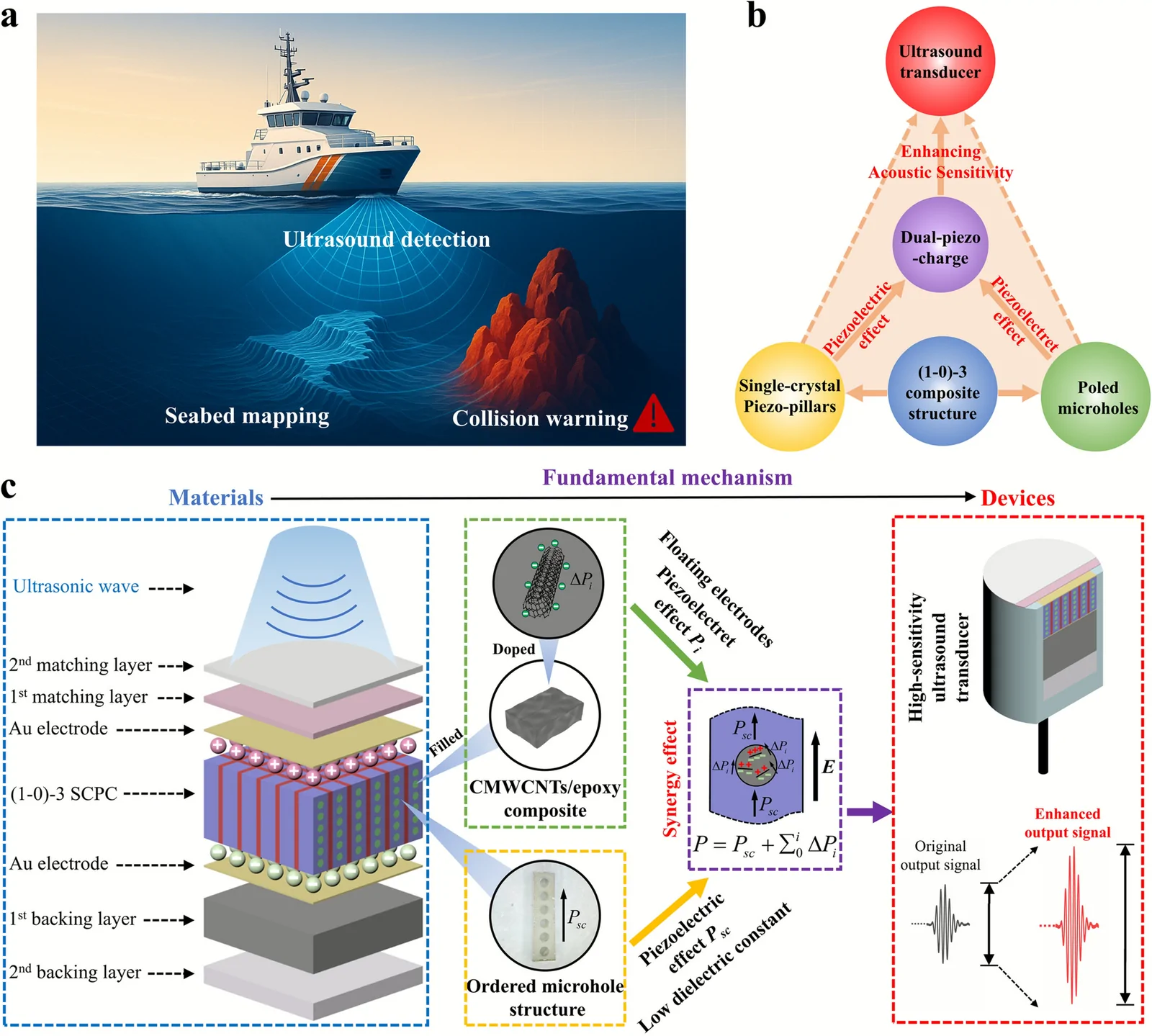
Schematic diagram of the enhanced acoustic sensing principle of a (1–0)–3 composite structure ultrasonic transducer. **a** Application scenario of ultrasound transducer for underwater mapping and collision avoidance. **b, c** Exploded schematic diagrams of the (1–0)–3 SCPC containing 0-phase polar microholes and the DPC mechanism underlying the performance enhancement of PUT

Figure 1 reveals perceptual and conceptual diagrams of the proposed PUT working underwater and exploded schematic diagrams of the (1–0)–3 SCPC and the fundamental mechanism of DPC underlying the performance enhancement. Figure 1b illustrates the relationship among the (1–0)–3 composite structure and PUT with enhanced sensitivity, including the DPC mechanism inside the composite structure: (i) the piezoelectric effect contributed by the aligned single-crystal piezo-pillars and (ii) the piezoelectret effect contributed by the ordered and poled microholes. As shown in Fig. 1c, the ordered 0-phase microholes structure within the piezoelectric pillars significantly reduces the dielectric constant and mass density of the composite material. Simultaneously, CMWCNTs are uniformly dispersed in the epoxy resin, acting as floating electrodes in microholes. Under the influence of the strong direct current electric field, the excess net charges are induced on floating electrodes in microholes, forming micro-dipoles due to the dielectric constant difference between CMWCNTs and the epoxy resin in the 0-phase microholes. This phenomenon is often called as piezoelectret effect. Although the CMWCNTs are randomly distributed within the epoxy resin, the generated micro-dipoles along the polarization direction always contribute positively. Additionally, the applied electric field enhances the field penetration through the action of CMWCNTs, guiding the poling direction at the microhole edges, thereby creating more stable polarization regions and mitigating the detrimental effect of the pores on the polarization response. Figure S4 illustrates the potential mechanism underlying the enhancement of the polarization response of the piezoelectric single crystal by polar microholes. The unique DPC mechanism, stemming from piezoelectric and piezoelectret synergy effect, ultimately leads to an enhancement in the acoustic sensitivity of underwater ultrasonic transducers. High-sensitivity underwater acoustic detection enables vessels navigating seawater to detect submerged reefs at an early stage, thereby avoiding potential underwater collision accidents, which is extremely important for maritime safety.

Based on the hydrostatic response physical model of 1–3 piezocomposite [55], Text S4 provides a detailed analysis on how the volume fraction of the piezoelectric phase affects the piezocomposite properties. The piezoelectric coefficients, dielectric constants, FOMs, electromechanical coupling factors, and acoustic impedances of SCPCs with different volume fractions were calculated by using the COMSOL Multiphysics (COMSOL Inc., Sweden). As shown in Fig. S6a, the introduction of ordered microhole structure within the piezo-pillars significantly reduces the effective dielectric constant, while the decrease in the *d*_33_ is relatively small. Therefore, as shown in Fig. S6c, the *d*_h_*g*_h_ of the (1–0)–3 SCPC, which is crucial to the design of piezocomposites for high-sensitivity ultrasonic transducer, is significantly enhanced. When the volume fraction of the piezoelectric phase is 40%, the *d*_h_*g*_h_ shows a 110% increase compared to that of conventional 1–3 piezocomposites. Meanwhile, due to the removal of a portion of the piezoelectric material in the piezo-pillars, the thickness electromechanical coupling coefficient *k*_t_ decreases slightly, but remains at a relatively high level (~ 78%), while the poled microholes generate an additional piezoelectric or force–electric response due to piezoelectret effect, which ensures higher receiving sensitivity.

**Fig. 2:**
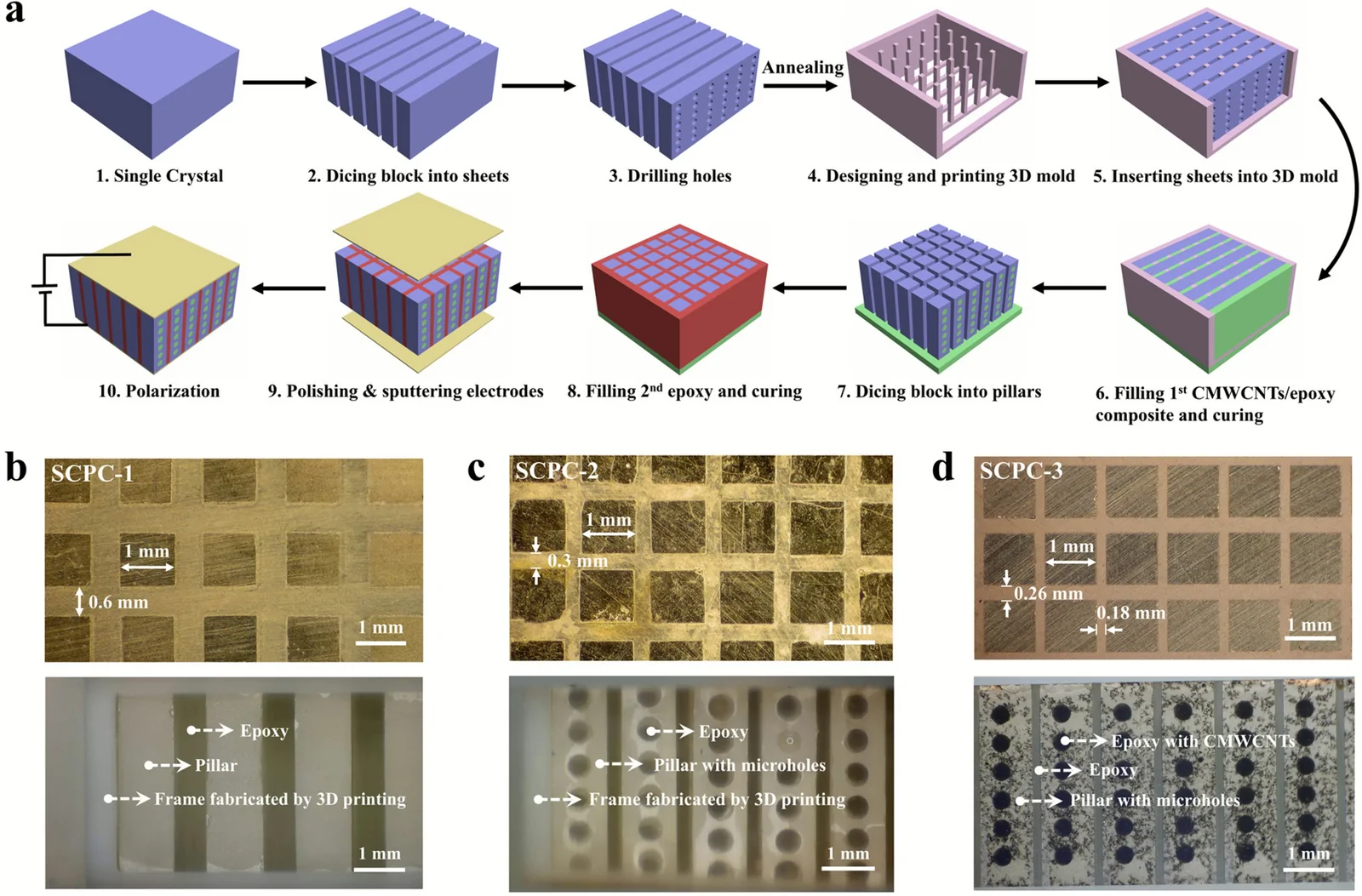
Fabrication process and optical images of the composite material. **a** Diagram of fabrication processes of (1–0)–3 SCPC featuring polar 0-phase microholes. Optical image of **b** 1–3 SCPC, **c** (1–0)–3 SCPC with nonpolar microholes, and **d** (1–0)–3 SCPC

To ensure consistency, the sufficient number of single-crystal plates was cut from the same wafer for the fabrication of three different types of SCPCs. The piezoelectric coefficients of the single-crystal plates were measured in advance, and those with inferior piezoelectric performance were excluded. This property inhomogeneity is difficult to avoid during single-crystal growth and is a great challenge for the preparation of SCPCs with good consistency. Three types of SCPCs were fabricated based on [011]-oriented relaxor PIN-PMN-PT crystal via a modified method combining 3D printing-assisted dice-and-insert with dice-and-fill techniques, and the entire fabrication process of (1–0)–3 SCPC is shown in Fig. 2a. The ordered array of microholes was first precisely fabricated on the single-crystal plates using a diameter bronze grinding head. Subsequently, an annealing process was performed to relieve any residual mechanical stress in the single-crystal plate and to prevent crack formation during subsequent processing. The annealing process parameters are presented in Fig. S7b, and the fabricated microhole structure is shown in Fig. S7c. Single-crystal plates were cleaned with acetone, isopropyl alcohol, and deionized water and dried at 65 °C for 1 h, and the pillars were then positioned into a custom-designed 3D-printed mold with high dimensional accuracy, as shown in Fig. S7f. The prepared CMWCNTs/epoxy composites were filled into gaps, and then, samples were cured at room temperature for 24 h. After curing, the samples were diced into 1 × 1 × 4 mm^3^ piezoelectric pillars using a dicing machine, with the substrate retained and the CMWCNTs/epoxy composite confined to the microhole regions. Following cleaning and drying, the prepared epoxy was backfilled into the kerfs after vacuum degassing and cured at room temperature. Both sides of the SCPC were carefully polished with sandpaper to expose the single-crystal pillars while avoiding exposure of the microhole structure. After a second round of cleaning and drying, the gold electrodes with a thickness of 200 nm were sputtered onto the top and bottom surfaces of the samples by magnetron sputtering. Finally, the (1–0)–3 SCPC is polarized under a strong electric field with an amplitude of 10 kV cm^−1^ for 5 min. The detailed fabrication procedures for both the 1–3 SCPC and the 1–3 SCPC with nonpolar microholes are provided in Text S5.

Figure 2b–d presents the schematic diagrams of three types of SCPCs, highlighting the differences in their structural configurations. Figure 2b shows a schematic structure of a conventional 1–3 SCPC (SCPC-1), which is used for comparison. Figure 2c demonstrates the 1–3 composite structure with nonpolar microholes (SCPC-2), by introducing the nonpolar microholes structure within single-crystal pillars. As these microholes were filled with pure epoxy resin, they remain non polar even under high electric fields. Figure 2d illustrates the (1–0)–3 structure featuring polar 0-phase microholes (SCPC-3). In contrast to the nonpolar microholes in SCPC-2, the 0-phase microholes were filled with an epoxy composite doped with 0.25 wt% CMWCNTs. Under high electric fields, this configuration induces a piezoelectret effect, exhibiting polarization behavior. The CMWCNTs/epoxy composites were selectively filled into the microholes within the single-crystal pillars. This design avoids to fill the inter-pillar regions with CMWCNTs/epoxy composites, as the high modulus of CMWCNTs/epoxy composites will reduce the piezocomposite's compliance and impose a stronger clamping effect on the single-crystal pillars, which in turn degrades the *k*_t_. The CMWCNTs were dispersed within the epoxy matrix and function as floating electrodes. During poling, the dielectric mismatch between CMWCNTs and epoxy leads to interfacial charge accumulation, resulting in a piezoelectret effect occurring. And the CMWCNTs can improve the local electric field distribution near the microhole edges, promoting more stable polarization regions and mitigating the adverse impact of microholes on polarization response.

**Fig. 3:**
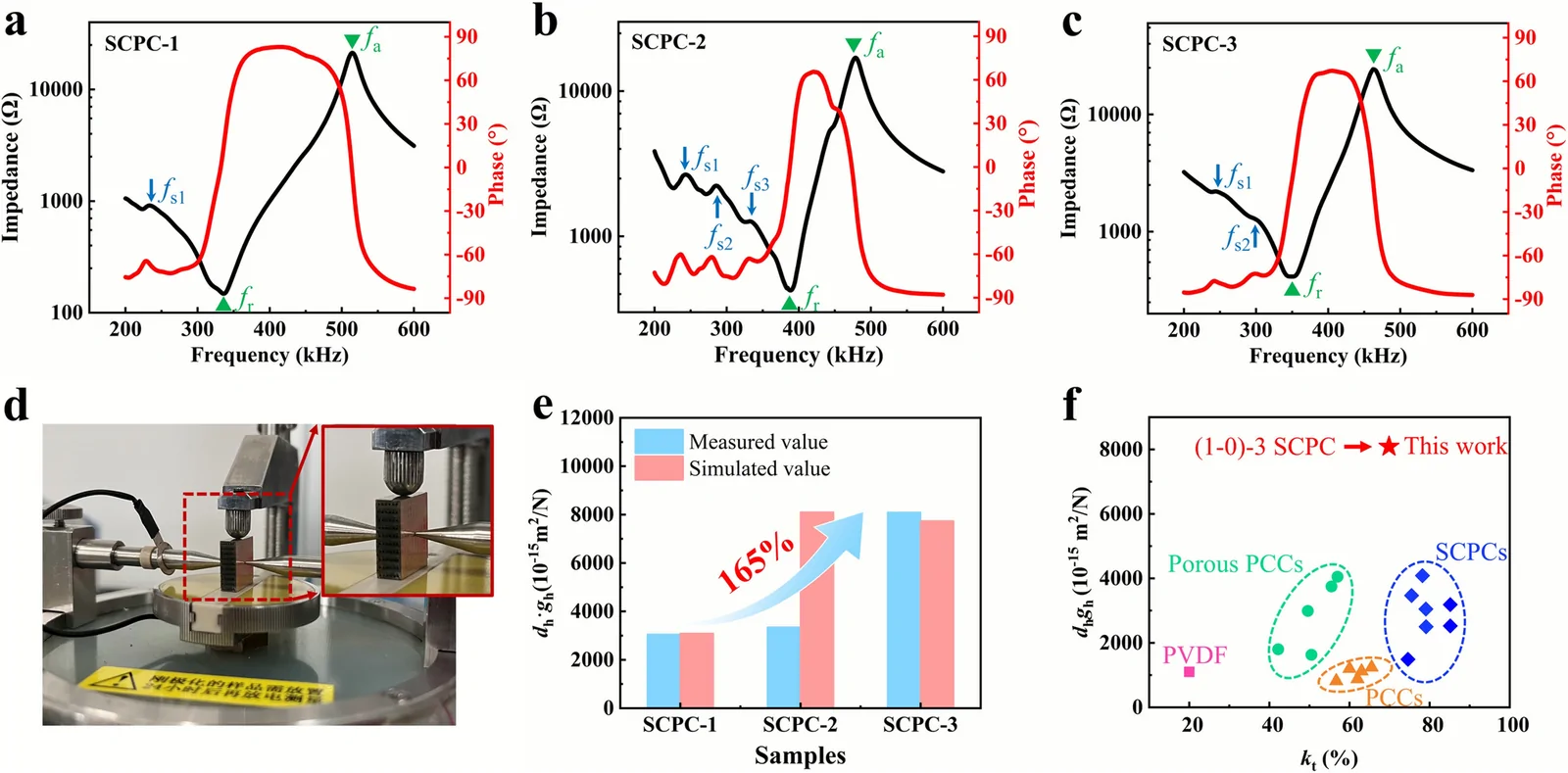
Electromechanical performance characterization of SCPCs. Impedance and phase spectra of **a** 1–3 SCPC, **b** (1–0)–3 SCPC with nonpolar microholes, and **c** (1–0)–3 SCPC. **d** Optical photograph of the experimental fixture for measuring *d*_31_/*d*_32_ piezoelectric coefficients. **e** Comparison between experimental and simulation results of the *d*_h_*g*_h_​ for three types of SCPCs. **f** Comparison of the *d*_h_*g*_h_ and *k*_t_ values with previously reported PVDF film [56], PCCs [24, 36, 57–59], SCPCs [24, 36, 60], porous PCCs [43, 54, 59, 61], and the (1–0)–3 SCPC in this work

Figure 3a–c shows the impedance and phase spectrum of three SCPCs in air. All three samples with different structures exhibit pure thickness longitudinal vibration mode as the primary working mode over a wide frequency range. The *k*_t_ of the three SCPCs is calculated to be 79.1%, 65.6%, and 69.8%, respectively. The SCPC-1 shows the maximum *k*_t_ value, benefiting from the high longitude electromechanical coupling coefficient *k*_33_ of tall pillars with square cross section. In the SCPC-2, the presence of microhole structures and the potential incomplete polarization of some piezoelectric single-crystals contribute to a reduction in *k*_t_. However, the CMWCNTs doping mitigates the adverse effect of microholes on polarization response. As a result, the SCPC-3 exhibits a higher *k*_t_​ value compared to SCPC-2. Additionally, all three impedance spectra exhibit a redundant mode, which is attributed to the uncoupled lateral vibration of the single-crystal pillars and is characterized by its anti-resonance frequency *f*_s1_. The frequency of this uncoupled lateral vibration mode can be tuned by altering the cross-sectional dimensions of the single-crystal pillars [24]. Furthermore, additional redundant modes are observed before the primary resonance frequency *f*_r_ in both the SCPC-2 and SCPC-3, due to the presence of microholes within the single-crystal pillars. However, fewer and weaker redundant modes are observed in the (1–0)–3 composite, which is attributed to the stronger constraint effect on the microhole-induced redundant modes exerted by the high-modulus CMWCNTs/epoxy composite. Attenuation of undesired modes adjacent to the primary resonance facilitates the enhancement of bandwidth in ultrasonic transducers.

Figure 3d shows a specially designed fixture that enables direct measurement of the *d*_31_​ and *d*_32_​ coefficients of the SCPCs using a *d*_33_​​ meter. Further information on the measurement and calculation of other properties is provided in Text S1. Figure 3e presents a comparison of the measured and simulated *d*_h_*g*_h_ coefficient for the three fabricated SCPCs. A substantial enhancement in the *d*_h_*g*_h_ is observed for the (1–0)–3 SCPC compared to the conventional 1–3 SCPC, with an increase of 165.2%.

Table 1 summarizes the key electrical and acoustic properties of the three fabricated SCPCs as characterized in this study. It should be noted that although the 1–3 composite with nonpolar microholes exhibits an abnormally high *d*_h_*g*_h_​ value in simulations, the experimentally measured value is significantly lower due to incomplete polarization of the piezoelectric single crystal caused by the presence of microholes. For the (1–0)–3 SCPC, the polar microholes effectively alleviate the negative impact of porosity on the polarization behavior and provide the additional piezoelectret effect, thereby enabling a high *d*_h_*g*_h_ value of 8088.9 × 10^−15^ m^2^ N^−1^. It is worth noting that, as shown in Fig. 3f, the (1–0)–3 SCPC achieves a maximum *d*_h_*g*_h_, which significantly surpasses those of the other representative state-of-the-art piezoelectric composites, including PVDF film [56], piezoceramic composites (PCCs) [24, 36, 57–59], SCPCs [24, 36, 60], and porous PCCs [43, 54, 59, 61]. The *d*_h_*g*_h_ obtained from (1–0)–3 SCPC is approximately 443.2% higher than that of commercial 1–3 SCPC [36] and 890.6% higher than commercial 1–3 PCC [24], respectively, indicating the great potential of the novel (1–0)–3 piezocomposite for improving the hydrostatic performance of ultrasonic transducers.

**Table 1:** Properties of the fabricated SCPCs

	*f*_*r*_ (kHz)	*f*_a_ (kHz)	*Ρ *(kg m^−3^)	*d*_33_ (pC N^−1^)	$\varepsilon_{33}^{{\mathrm{T}}} /\varepsilon_{0}$	*d*_h_ (pC N^−1^)	*g*_h_ (mV mN^−1^)	*d*_h_*g*_h_ (10^–15^ m^2^ N^−1^)	*k*_t_ (%)	*Z* (MRayl)
1–3 SCPC	333	512	4352.3	914	1486.4	200.3	15.23	3049.8	79.1	17.2
1–3 SCPC with nonpolar microholes	384	488	4491.1	499	852.7	159	21.07	3350.1	65.6	15.9
(1–0)–3 SCPC	348	464	4531.8	726	951.6	261.4	30.94	8088.9	69.8	16.7

### Fabrication and Characterization of (1–0)–3 SCPC-based PUT

**Fig. 4:**
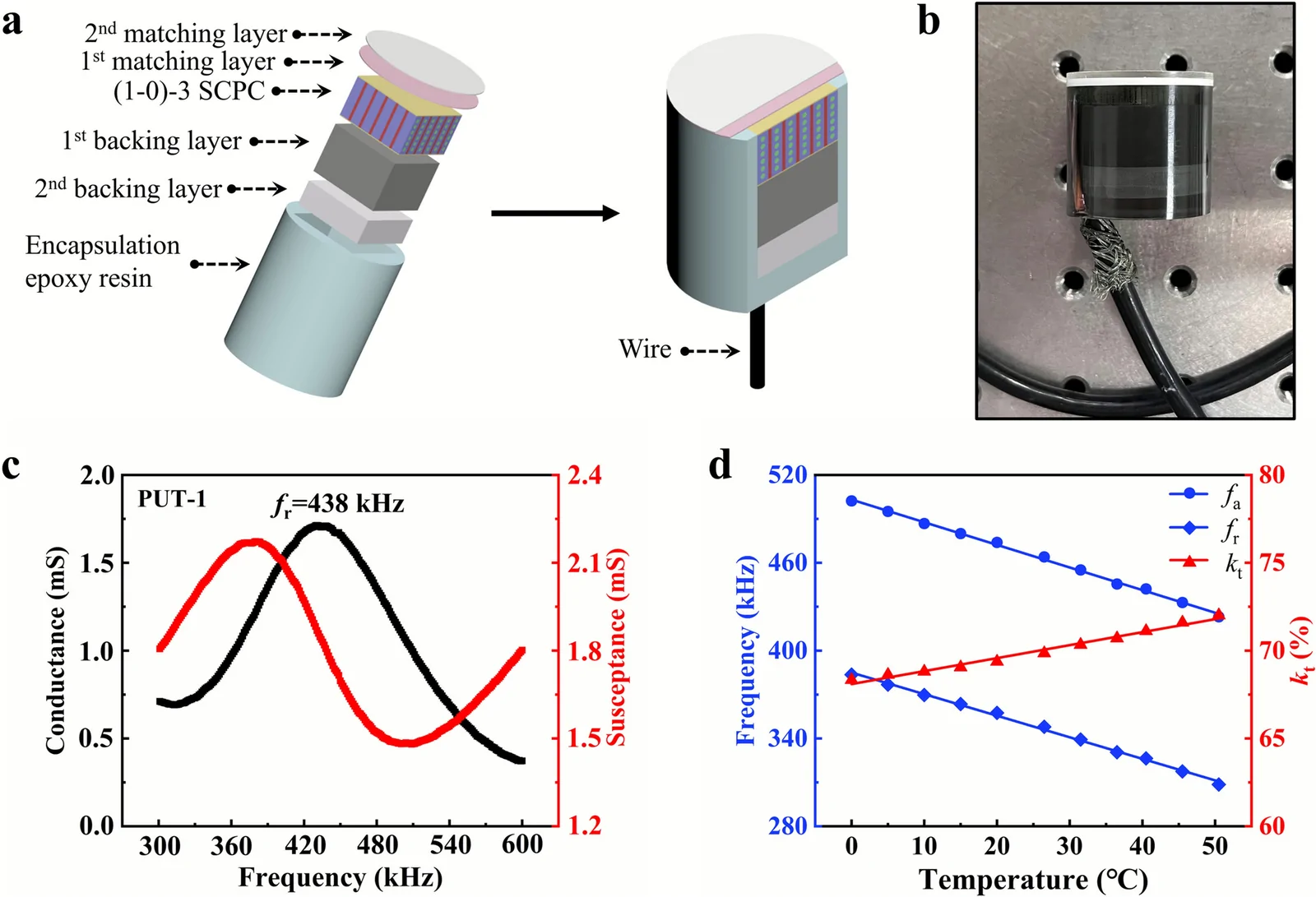
Schematic of the PUT based on (1–0)–3 SCPC and its corresponding frequency characteristics. **a** Schematic illustration of the (1–0)–3 SCPC-based PUT with key components labeled. The SCPC is embedded in epoxy matrix. The matching layers and backing layers are bonded to the top and bottom surfaces, respectively. **b** Optical image of the fabricated PUT-1. **c** Conductance and susceptance spectra in water of PUT-1. **d** Temperature dependence of resonant frequency *f*_r_, anti-resonant frequency *f*_a_, and thickness electromechanical coupling coefficient *k*_t_ of the (1–0)–3 SCPC

An ultrahigh-sensitivity ultrasonic transducer with the acoustic matching layers and backing layers for effective transmitting/receiving sound waves was further fabricated based on the (1–0)–3 SCPC. A diagram of the as-designed (1–0)–3 SCPC-based PUT, denoted as PUT-1, is shown in Fig. 4a. Besides the SCPC as the core functional element, PUT-1 comprises two acoustic matching layers, two backing layers, and epoxy resin for waterproofing. Text S7 provides a detailed description of the design methodology and fabrication process of the acoustic matching layers and backing layers. As shown in Fig. S10, to ensure good adhesion between components, partial electrodes were fabricated on the side surfaces of the SCPCs using a shadow mask. The conductive silver epoxy was used to secure the wires to the side surfaces of the SCPCs. Subsequently, the matching and backing layers were bonded to the top and bottom surfaces of the (1–0)–3 SCPC, respectively. Finally, the PUT-1 was successfully fabricated following epoxy encapsulation, which provides mechanical protection and waterproofing. For comparison, the conventional 1–3 SCPC was also assembled into a PUT, referred to as PUT-2.

Figure 4b shows the PUT-1, which is ready for direct experimental testing. Initially, the conductance and susceptance spectra of the PUT-1 were measured underwater at a depth of 0.5 m to assess its performances in a practical environment. As shown in Fig. 4c, the conductance spectrum of PUT-1 exhibits a single peak over a broad frequency range, which is favorable for the device's performance underwater. In addition, when the PUT operates in real environments, temperature variation is a key factor that needs to be carefully considered. In this work, to investigate its temperature stability, an impedance analyzer (E4990A, Keysight Technologies, USA) and a high-temperature dielectric impedance spectrometer (DMS1000, Balab Technologies, China) were used to experimentally determine the impedance characteristics of the novel (1–0)–3 SCPC in the temperature range of 0 to 50 ℃. Figure 4d shows the temperature dependence of *f*_r_, *f*_a_ and *k*_t_ of (1–0)–3 SCPC. Both *f*_r_ and *f*_a_ decrease nearly linearly as the temperature rises from 0 to 50 °C, while *k*_t_ increases from 68.3% to 72%. The phenomenon arises from the reduction in the elastic modulus of the epoxy resin as temperature increases, weakening its clamping on the piezoelectric pillars [62, 63]. Simultaneously, the piezo-pillars show a stronger mode of longitudinal vibration, resulting in a higher *k*_t_. To better benchmark this results against conventional 1–3 composite, the temperature dependence of *f*_r_, *f*_a_ and *k*_t_ of the 1–3 SCPC is presented in Fig. S8. Within the typical ocean temperature range (0–30 °C), the center frequency of the (1–0)–3 SCPC shifts upward by approximately 37 kHz, while the variation in *k*_t_ remains within about 2.3%. In contrast, for the 1–3 SCPC, these two parameters change by 29 kHz and 2.9%, respectively. The (1–0)–3 SCPC shows a slightly higher frequency drift with temperature than the conventional 1–3 SCPC. This phenomenon is attributed to the microholes effect in the (1–0)–3 SCPC, resulting in more pronounced lateral deformation as temperature variation. Overall, because the two composites have very similar volume fractions of the piezoelectric single crystal, they exhibit essentially comparable temperature stability.

**Fig. 5:**
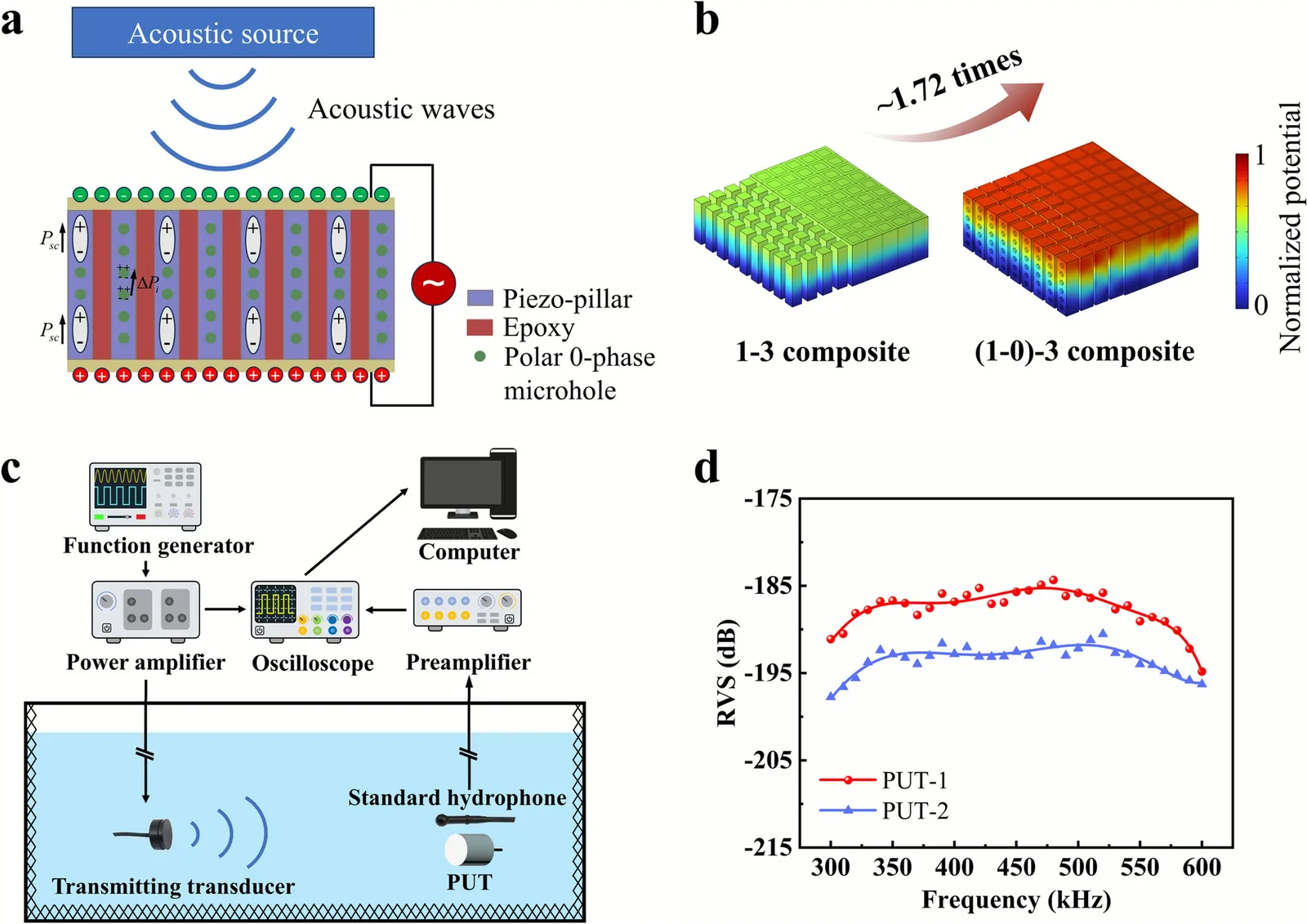
Measurement of the receiving sensitivity of the PUTs. **a** Working principle of PUTs. **b** Comparison of the simulated normalized electric potential distributions of the two piezoelectric composites under an underwater acoustic field. **c** Schematic setup for measuring the receiving performance of the PUTs. **d** RVS curves of the PUTs

In practical applications, one of the primary functions of PUTs is to transform incoming ultrasonic waves into corresponding electrical signals. Figure 5a illustrates the operating principle of the PUT. The finite element analysis was conducted to carry out the electric potential output of the conventional 1–3 SCPC and the (1–0)–3 SCPC under hydrostatic conditions. Figure 5b shows the electric potential distributions of the two composites under the same acoustic pressure. It is worth noting that the overall equivalent parameters of the (1–0)–3 model were directly assigned based on the experimentally measured values of the SCPC-3. This simulation approach integrates the contributions of both the electret effect and the piezoelectric effect to the overall performance of the composite material, while also reducing the complexity and computational cost of the simulation. Under identical conditions, the (1–0)–3 SCPC exhibits an output voltage that is approximately 1.72 times higher than that of conventional 1–3 SCPC, indicating higher sensitivity. Figure 5c depicts the measurement system for characterizing the receiving performances of the fabricated PUTs. A calibrated transmitting transducer (RESON TC 3021, Denmark) serves as an ultrasonic energy source, submerged in a large anechoic tank. A calibrated standard hydrophone and fabricated PUTs were used to receive acoustic waves and generate output voltage signal at the 1 m distance from the transmitting transducer, respectively. All devices were positioned at a depth of 0.5 m. The receiving voltage sensitivity (RVS), which evaluates the sensitivity of the PUT, was finally calculated using the substitution method. The experimental procedure is described in detail in the Experimental Section. Figure 5d shows the RVS curves of the two PUTs. It can be observed that the PUT based on (1–0)–3 SCPC exhibits a higher overall receiving sensitivity, with a maximum RVS value of − 184.3 dB, compared to − 190.5 dB for the 1–3 SCPC-based PUT. In contrast, the RVS curve of PUT-2 exhibits the flattest frequency response over a broader frequency range, indicating a wider operational bandwidth. The − 3dB receiving frequency bandwidths of the two devices are 130 and 180 kHz, respectively. The RVS curves of the two transducers exhibit almost identical overall trends, while the detailed variations correspond well with the vibration mode characteristics of the respective piezocomposites. Although the (1–0)–3 SCPC exhibits more redundant vibration modes (corresponding to *f*_s1_ and *f*_s2_) before the *f*_r_ compared to the 1–3 SCPC, their relatively low intensities lead to minimal impact on the overall device performance. Previous studies have also demonstrated that uncoupled lateral vibration modes exert the limited influence on the dominant working mode [24]. Therefore, the working bandwidth of the device is primarily determined by the thickness electromechanical coupling coefficient *k*_t_ of the piezocomposite itself. Due to the higher *f*_a_ of the 1–3 SCPC, PUT-2 exhibits its maximum RVS at a higher frequency (520 kHz) compared to PUT-1 (480 kHz) and demonstrates a flatter performance response in the high-frequency range (500–580 kHz). The receiving performances of the two devices are detailed in Table 2.

**Table 2:** Receiving properties of the fabricated PUTs. *f*_1_, *f*_2_: the −3 dB frequency points

	RVS_max_ (dB)	*f*_1_ (kHz)	*f*_2_ (kHz)	*f*_c_ (kHz)	BW_-3dB_ (kHz)
PUT-1	−184.3	390	520	455	130
PUT-2	−190.5	370	550	460	180

**Fig. 6:**
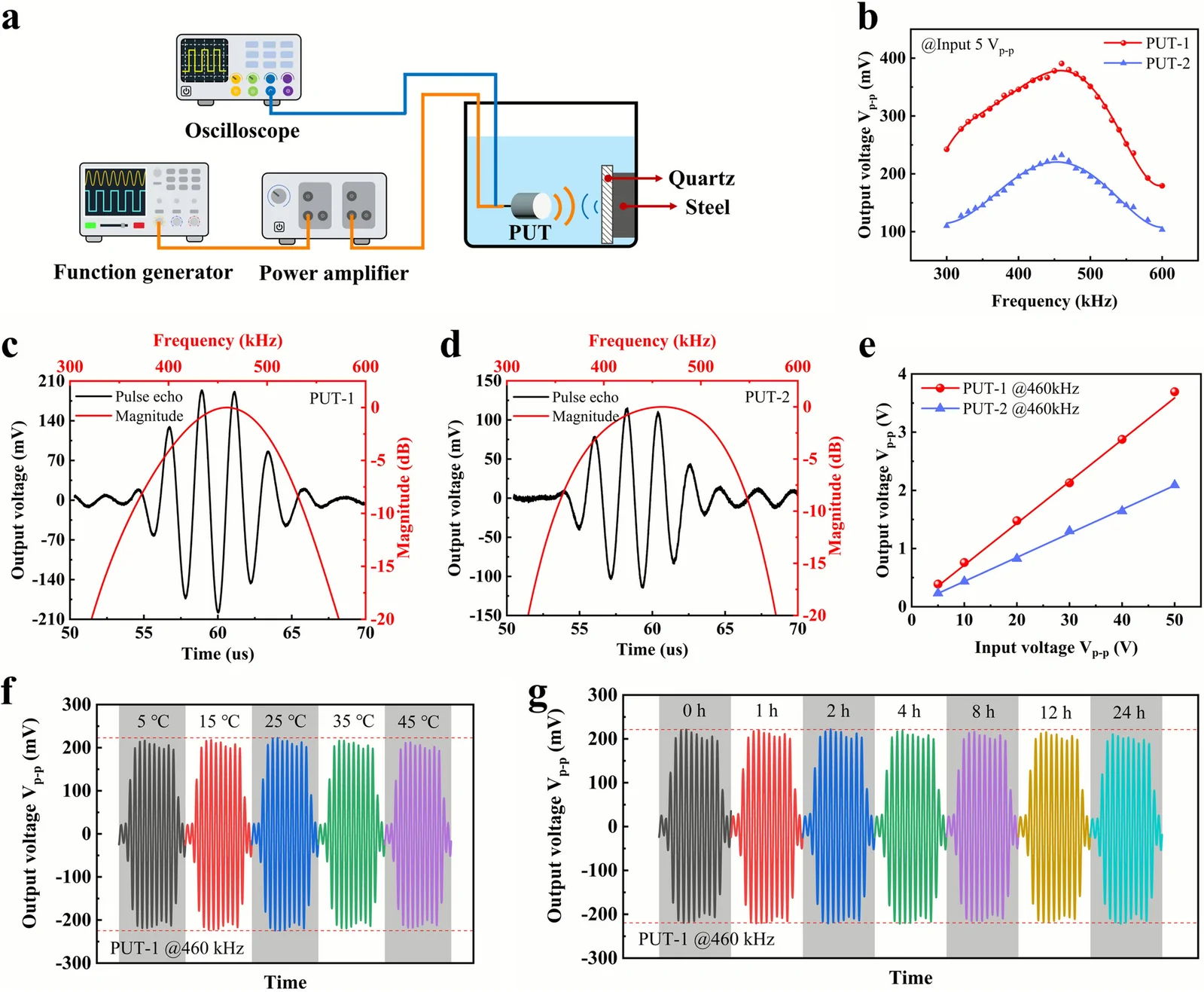
Experimental characterization of self-transmit-receive properties for the fabricated PUTs.** a** Schematic illustration of the experimental setup for testing the self-transmit-receive properties of the transducer. **b** Output voltage of the two PUTs as a function of frequency under a trigger voltage of 5 *V*_p-p_. Pulse-echo waveforms and frequency spectra for **c** PUT-1 and **d** PUT-2. **e** Output voltage of the two PUTs as a function of the trigger voltage at their respective optimum operating frequencies. **f** Self-transmit-receive performance of PUT-1 at different water temperatures. **g** Stability of PUT-1 output voltage over prolonged continuous operation

Furthermore, the self-transmit-receive performance was evaluated by conducting the pulse-echo experiment in a tank filled with ultrapure water. As illustrated in Fig. 6a, a sinusoidal pulse signal with three cycles was generated by an arbitrary function generator (AFG 31000, Tektronix, USA) to excite the fabricated transducer for underwater acoustic wave transmission. Subsequently, the transducer receives the acoustic waves reflected from the quartz plate and generates a corresponding voltage signal. During the experiment, the radiating surface of the transducer was carefully aligned parallel to the quartz plate to ensure maximum output voltage. The detailed experimental procedure is provided in the Experimental Section. Figure 6b presents the output voltage responses of the two PUTs as a function of frequency under a fixed input voltage. The optimal operating frequencies were determined by sweeping the input signal frequency, which was identified to be 460 kHz for both PUT-1 and PUT-2. At their respective optimal frequencies and under a 5 V peak-to-peak excitation, the maximum output voltage amplitudes for PUT-1 and PUT-2 were measured to be 390.7 and 232.4 mV, respectively, with PUT-1's maximum output voltage increasing by 68.1%. In addition, Fig. 6c, d presents the measured pulse-echo waveforms and corresponding frequency spectra of the two PUTs at their respective optimal operating frequencies. The − 3 dB bandwidths of PUT-1 and PUT-2 were measured to be 95 and 126 kHz, respectively. As shown in Fig. 6e, the output voltages of the two PUTs increase linearly with the input voltage, indicating good signal fidelity and stable transduction characteristics.

In addition, the temperature dependence of the PUT-1 was further investigated by evaluating the self-transmit-receive performance at various water temperatures. Figure 6f presents the pulse-echo waveforms of PUT-1 measured in water at temperatures of 5, 15, 25, 35, and 45 °C. A slight decrease in output voltage is observed as the operating temperature deviates from room temperature, with reductions of 2.6% at 5 °C and 3.9% at 45 °C. This attenuation is primarily attributed to the temperature-induced shift in the device’s optimal operating frequency. In practical applications, this issue can be effectively mitigated by dynamically adjusting the operating frequency in response to environmental temperature variations. Figure 6g displays the pulse-echo waveforms of PUT-1 during continuous operation for up to 24 h. Comparison of the output voltages at different operating durations shows that PUT-1 maintains essentially unchanged performance over the first 12 h of continuous operation. However, after 24 h of continuous working, a slight performance degradation was observed, with the output voltage decreasing by 1.93% compared to the initial value. This slight degradation in performance is likely attributable to electrical fatigue resulting from the repeated electric loading applied to the piezoelectric material [64]. These results demonstrate the excellent operational stability of the device under prolonged working conditions. Moreover, a comparison with previously reported devices [24] indicates that the introduction of the ordered microholes structure within the single-crystal pillar does not harm the overall stability of the device.

Finally, the performance of the PUT-1-based (1–0)–3 SCPC was compared with that of other reported PUTs [24, 25, 30, 31, 61, 65], as summarized in Table S4. Conclusively, the (1–0)–3 SCPC-based PUT demonstrates the enhanced output voltage sensitivity compared to the conventional 1–3 SCPC-based PUT and the previously reported transducers, while maintaining a broad − 3 dB bandwidth of 130 kHz. This work indicates that the proposed (1–0)–3 piezocomposite structure, along with the design strategy of incorporating 0-phase microholes forming micro-dipoles through filling CMWCNTs/epoxy resin, is promising for future ultrasonic transducer development.

## Conclusion

In summary, we report a design strategy for developing innovative (1–0)–3 SCPCs based on the [011]-oriented relaxor ferroelectric PIN-PMN-PT crystal, as well as a high-performance PUT with ultrahigh sensitivity. This study introduces ordered and polar microholes (0-phase) into piezoelectric single-crystal pillars (1-phase) composited with epoxy resin (3-phase) for the first time, resulting in a marked reduction in effective dielectric constant and mass density, along with improved acoustic receiving sensitivity. When 0.25 wt% CMWCNTs are introduced into the epoxy resin and a strong poling field is applied, the net charges at the interface between CMWCNTs and epoxy resin enable 0-phase microholes to be poled, forming micro-dipoles. Therefore, the 0-phase microholes exhibit the piezoelectret effect. Along with the improvement of the local electric field, the polar microholes significantly mitigate the negative impact of the microhole structure on the polarization response of the composite material. Moreover, the coupling between the piezoelectret effect of the polarized microholes and the intrinsic piezoelectric effect of the piezo-pillars results in a unique DPC mechanism. A modified method combining 3D printing-assisted dice-and-insert with dice-and-fill techniques is used to fabricate the innovative (1–0)–3 SCPC. Both simulation calculations and experiments have demonstrated that the innovative structure and unique DPC mechanism in (1–0)–3 SCPC can significantly enhance the *d*_h_*g*_h_ of the composite. The (1–0)–3 SCPC achieves a remarkable *d*_h_*g*_h_ of 8088.9 × 10^−15^ m^2^ N^−1^, representing increases of 165.2%, 443.2%, and 890.6% over a conventional 1–3 composite structure, a commercial 1–3 SCPC, and a commercial 1–3 PCC, respectively.

The PUTs based on (1–0)–3 SCPC and conventional 1–3 SCPC with matching layers and backing layers are fabricated and measured. Compared with PUT based on conventional 1–3 piezocomposite structure, the proposed PUT based on novel piezocomposites exhibits better receiving sensitivity due to the DPC mechanism. When used as a standalone receiver, the (1–0)–3 SCPC-based PUT achieves a maximum receiving voltage sensitivity (RVS) of − 184.3 dB, with a − 3 dB working bandwidth of 130 kHz. Meanwhile, in the case of active detection, the novel transducer demonstrates better receiving sensitivity, with an output voltage of 68.1% higher than that of the conventional 1–3 composite transducer under the same excitation conditions. Clearly, an enhanced underwater acoustic sensing is extremely important for maritime safety, avoiding potential underwater collision hazard. Additionally, prolonged working has demonstrated the long-term stability of the SCPC transducer containing polar 0-phase microholes. Therefore, this design strategy is meaningful, which introduces the order and polar microhole structure in single crystal and utilizing the DPC mechanism to enhance the underwater acoustic sensitivity of ultrasonic transducers. Meanwhile, the underlying principle—synergistic contribution of multiple piezoelectric charge-generating mechanisms—can, in principle, be extended to other piezoelectric crystal systems or composite architectures, provided that appropriate structural design and polarization strategies are employed. This work also provides guidance for the development and improvements of next-generation piezocomposite materials and broadband, high-sensitivity ultrasonic transducers as well.

## Supplementary Information

Below is the link to the electronic supplementary material.

Supplementary file1 (DOCX 3082 KB)
