# Supplementary material for: Dual-Piezo-Charge Strategy in (1–0)–3 Single-Crystal Composite for Enhancing Underwater Acoustic Sensing
Source: Nanomicro Lett. 2026 Feb 28;18:259. doi: 10.1007/s40820-026-02102-1 (PMC12950123; doi:10.1007/s40820-026-02102-1)
Supplement: Supplementary file 1 — Supplementary file1 (DOCX 3082 KB) [file 40820_2026_2102_MOESM1_ESM.docx]

Supporting Information for

**Dual-Piezo-Charge Strategy in (1-0)-3 Single-Crystal Composite for Enhancing Underwater Acoustic Sensing**

Yu Lei^1,2^, Xiaotian Li^3^*, Zewei Hou^1,4^, Bing Wang^5^, Shuguang Zheng^6^, Yang Wei^7^, Zhonghui Yu^2^, Jiawang Hong^1,4,8^*, Shuxiang Dong^2^*

^1^ School of Aerospace Engineering, Beijing Institute of Technology, Beijing 100081, P. R. China

^2^ School of Materials Science and Engineering, Peking University, Beijing 100871, P. R. China

^3^ College of Semiconductors (College of Integrated Circuits), Hunan University, Changsha 410082, P. R. China

^4^ Beijing Institute of Technology, Zhuhai 519088, P. R. China

^5^ Institute for Advanced Study, Shenzhen University, Shenzhen 518061, P. R. China

^6^ Electronic Materials Research Laboratory, Key Laboratory of the Ministry of Education and International Center for Dielectric Research, School of Electronic Science and Engineering, Xi’an Jiaotong University, Xi’an 710049, P. R. China

^7^ College of Mechanical and Electrical Engineering, Hohai University, Changzhou 213200, P. R. China

^8^ State Key Laboratory of Environment Characteristics and Effects for Near-space, Beijing institute of technology, Beijing, 100081, PR China

*Corresponding authors. E-mail: [lixiaotian@hnu.edu.cn](mailto:lixiaotian@hnu.edu.cn) (Xiaotian Li); [hongjw@bit.edu.cn](mailto:hongjw@bit.edu.cn) (Jiawang Hong); [sxdong@pku.edu.cn](mailto:sxdong@pku.edu.cn) (Shuxiang Dong)

**S1** **Critical Parameters of 1-3, (1-0)-3 Single-crystal Piezoelectric Composites (SCPCs)**

In this work, the *d*_33_ of the SCPCs was determined as the average of 15 randomly selected measurement points, and the *d*_31_, *d*_32_ were obtained by averaging 7 random measurement points, respectively.

$d_{33}=\sum_{i}^{15} d_{33}^{i}/15$ (S1)

$d_{32}=\sum_{i}^{7} d_{32}^{i}/7$ (S2)

$d_{31}=\sum_{i}^{7} d_{31}^{i}/7$ (S3)

And the distribution of measurement points is shown in the Fig. S1 below to ensure that the piezoelectric coefficients of all regions of the sample are measured and statistically analyzed. The raw data of the *d*_3j_ (j=1, 2, 3) for the three SCPCs are presented in the Table S1.

The thickness electromechanical coupling coefficient kt of piezoelectric composites is primarily determined by (i) the electromechanical coupling factor *k*_33_ of the piezoelectric materials and (ii) the composite structure formed by the pillar array within the polymer matrix. A high *k*_t_ indicates efficient energy conversion and a broad operational bandwidth, and it can be expressed by the following formula:

$k_{t}=\sqrt{\frac{\pi f_{r}}{2f_{a}}\tan\frac{\pi\left(f_{a}-f_{r}\right)}{2f_{a}}}$ (S4)

where *f*_r_ and *f*_a_ are the resonant frequency and anti-resonant frequency in thickness vibration mode, respectively, which can be obtained from the impedance spectrum. Typically, in 1-3 composites, the dominant vibration of each piezoelectric pillar occurs along the thickness direction, resulting in a high *k*_t_​ although confined by the epoxy resin.

The relative constant stress permittivity $\varepsilon_{33}^{\text{T}}/\varepsilon_{0}$ of the piezocomposites is related to the dielectric property of material and its polarization state. Low $\varepsilon_{33}^{\text{T}}/\varepsilon_{0}$ is beneficial to improve the receiving sensitivity of the transducer.

$\varepsilon_{33}^{\text{T}}/\varepsilon_{0}=Ct/\varepsilon_{0}A$ (S5)

where $\varepsilon_{0}$, $C$, $t$, $A$ are the permittivity of vacuum, capacitance, thickness, and electrode area of the sample, respectively.

The piezoelectric voltage coefficient *g*_3j_ (j=1, 2, 3) determining the reception performances of piezoelectric composites can be calculated according to the known parameters *d*_3j_ and $\varepsilon_{33}^{\text{T}}$. Typically, the larger the *g*_33_, the higher the receiving sensitivity of the ultrasonic transducer. Therefore, a high *d*_33_ and low dielectric constant are essential.

$g_{3j}=d_{3j}/\varepsilon_{33}^{\text{T}}$ (S6)

The acoustic impedance *Z* of piezoelectric composites or ultrasonic transducers determines the energy transmission of the sound waves when passing through the medium interface from the transducer into the target medium (water or human tissue). A high *Z* often causes mismatching with water, leading a poor sensitivity of ultrasonic transducers. *Z* can be given by following formula:

$Z=\overline{\rho}\overline{v}$ (S7)

where $\overline{\rho}$ is equivalent density of the piezocomposites, $\overline{v}$ is the longitudinal sound velocity in composites. The measurement process of the longitudinal wave velocity of the sample is illustrated in Supplementary Information S6.

The figure of merits (FOMs) of piezoelectric composites, including *d*_33_*g*_33_ and *d*_h_*g*_h_, characterizes the receiving sensitivity at the material level, normally they indicate the receiving sensitivity when operating in 33 mode and hydrostatic mode, respectively. The ultrasonic transducer is in a hydrostatic pressure working mode underwater, therefore, the *d*_h_*g*_h_ parameter is critical. They can be given by following formula:

$d_{33}g_{33}=d_{33}^{2}/\varepsilon_{33}^{\text{T}}$ (S8)

$d_{h}g_{h}=d_{h}^{2}/\varepsilon_{33}^{\text{T}}$ (S9)

$d_{h}=d_{31}+d_{32}+d_{33}$ (S10)

where $d_{h}$ is the hydrostatic charge coefficient.

**S2 Effect of Microhole Structure on the Properties of SCPCs**

The simulations of the critical parameters and vibration modal deformation of microholes-contained (1-0)-3 SCPC are carried out using the piezoelectric module in COMSOL Multiphysics. The piezoelectric coefficients *d*_3j_ (j=1, 2, 3) are 708, -1695, 1271 pC/N, respectively, according to the actual measurement results, and other material coefficients of single crystal are obtained from previous literature [S1]. The main material parameters of the used epoxy resin for simulation are listed in Table S2. In this study, the dimension of all piezoelectric single-crystal pillars is fixed at 1×1×4 mm3 for simulation, ensuring that the width-to-thickness aspect ratio of the piezoelectric pillar is less than 0.5. To reduce computational cost, we established only a representative unit to predict the performance of the composite, with periodic boundary conditions applied. Firstly, the effect of microhole distribution on the single-crystal pillar on the overall performance of the composite material is analyzed. Based on the piezoelectric composite material series model, both $\overline{d_{33}}$ and $\overline{\varepsilon_{33}}$ will decrease significantly upon the introduction of a very thin layer with low permittivity. They can be given by following formula:

$\overline{d_{33}}=\frac{v^{c}d_{33}^{c}\varepsilon_{33}^{p}+v^{p}d_{33}^{p}\varepsilon_{33}^{c}}{v^{c}\varepsilon_{33}^{p}+v^{p}\varepsilon_{33}^{c}}$ (S11)

$\overline{\varepsilon_{33}}=\frac{\varepsilon_{33}^{c}\varepsilon_{33}^{p}}{v^{c}\varepsilon_{33}^{p}+v^{p}\varepsilon_{33}^{c}}$ (S12)

where $\overline{d_{33}}$ and $\overline{\varepsilon_{33}}$ represent the effective properties of the composite material, *v* is the volume fraction, and the superscripts *c* and *p* represent the piezoelectric phase and the polymer phase, respectively.

For a single-crystal pillar containing only one microhole, the spatial position of the microhole does not additionally affect the performance of the composite. However, when the number of microholes increases to two or more, their spatial distribution becomes a critical factor that significantly influences the composite’s performance. As shown in Fig. S2b, two distinct microholes configurations are investigated, namely, alignment parallel and perpendicular to the polarization direction of the crystal pillar. The simulation results reveal that the composite exhibits enhanced overall performance when the microholes are aligned parallel to the polarization direction. Conversely, when the microholes are oriented perpendicular to the polarization direction, their influence on the macroscopic piezoelectric response becomes more pronounced, as predicted by the series model of composite materials. Due to the inherent anisotropy of single-crystal, this study further explores the influence of microhole orientation on the composite's performance. As illustrated in Fig. S2c, three representative microhole orientations are analyzed and compared with a pore-free single-crystal composite. The normalized values of all critical parameters are presented in Fig. S2c. It can be observed that when the microhole orientation is along the 3- direction, the basic structure of the composite remains fundamentally unchanged. The distribution of the single-crystal pillar and epoxy resin follows the parallel model (Fig. S2a), resulting in negligible changes in the *d*_33_ and dielectric constant under the same volume fraction. When the microholes are oriented along the 1- and 2- directions, this introduces a partial series structure into the originally parallel 1-3 composite, leading to a significant reduction in the composite's effective dielectric constant. Furthermore, when the microholes are aligned along the 1- direction, the composite demonstrates a more favorable hydrostatic piezoelectric coefficient. The effect of microhole quantity on the overall performance of composite materials was further investigated with the same volume fraction. Fig. S2d presents a normalized values analysis of critical parameters for composite materials with three different microhole quantities compared to a microhole-free structure. Under the same volume fraction, an increase in the number of microholes corresponds to a reduction in their size. As a result, the impact of the microholes, acting as a series structure, on the overall performance becomes smaller. Therefore, theoretically, the more microholes present, the greater the *d*_33_ and hydrostatic figure of merit *d*_h_*g*_h_ of the piezocomposite. Ultimately, considering the trade-off between microhole fabrication difficulty and performance enhancement, a structure with 6 microholes and their diameter of 0.4 mm is selected in this work.

**S3** **Effect of Carboxylated Multi-walled Carbon Nanotubes (CMWCNTs) on the Performance of Composite Materials**

In practical experiments, the piezoelectric response of 1-3 composites with nonpolar microholes is often significantly lower than the results predicted by simulations. This discrepancy arises because the presence of microholes negatively affects the polarization response of the single-crystal pillars, leading to incomplete polarization in some piezoelectric crystals. In contrast, simulations typically assume that all single-crystal materials exhibit ideal piezoelectric behavior. Previous studies have demonstrated that multi-walled carbon nanotubes, when dispersed as a third phase in composite materials, can effectively enhance their dielectric and piezoelectric properties [S2–S5]. In this work, to mitigate the adverse effects of microholes on the polarization response of the piezocomposite, CMWCNTs were uniformly dispersed in epoxy resin and employed as a filler to occupy the microholes. The CMWCNTs used in this work have an inner diameter of 3–5 nm, an outer diameter of 8–15 nm, and a length of 5–15 μm. We first investigate the influence of dispersing CMWCNTs at various mass ratios in epoxy resin on the electrical properties. Fig. S3a illustrates the composite materials prepared by dispersing CMWCNTs with mass ratios of 0.1, 0.25%, 0.5%, and 1% into epoxy resin. Gold electrodes were fabricated on the top and bottom central surfaces of the composite materials (containing with 0.1 wt.% and 0.25 wt.% CMWCNTs) by using a vacuum sputtering method. The fabricated CMWCNTs/epoxy composite materials were subjected to a 1 kV/mm electric field in silicone oil for 5 minutes, which serves as the polarization condition for the SCPCs. Experimental results show that composites with 0.5 wt.% and 1 wt.% CMWCNTs suffer breakdown under the electric field, while those with 0.1 wt.% and 0.25 wt.% do not. Therefore, to prevent breakdown of the (1-0)-3 piezocomposite during polarization, the mass ratio of CMWCNTs is controlled to be 0.25%. It is worth noting that the dispersion of CMWCNTs in epoxy resin also significantly affects the electrical properties of the composite materials. As shown in Fig. S3b, even if the mass fraction of CMWCNTs is within a safe range (0.25 wt.%), aggregation of the CMWCNTs can occur if they are not evenly distributed in the epoxy resin, leading to the formation of conductive paths and increasing the likelihood of breakdown. Therefore, in preparing the CMWCNTs/epoxy composites, the CMWCNTs are incorporated into the epoxy matrix and centrifuged under vacuum at 1000 rpm for 12 minutes to achieve sufficient dispersion.

To intuitively demonstrate the improved polarization response induced by CMWCNTs, multiphysics coupling simulations were conducted using COMSOL software. All geometric dimensions of the single-crystal pillar were kept consistent with the actual samples. The polarization degree of the single-crystal pillar under the polarization field is shown in Fig. S4. In all the result figures, the colored areas represent regions that were successfully polarized (where the field strength exceeds the coercive field), while the white areas indicate regions that were not successfully polarized. Fig. S4a, 4b show the polarization response of the single-crystal pillars with microhole structures filled with air and epoxy resin with an electric field strength of 1 kV/mm, respectively. Due to the low and similar relative dielectric constants of both air and epoxy resin (~1 and 4, respectively), the volume fraction of the single-crystal that can be polarized in both structures is nearly identical, at 76.88% and 76.32%, respectively. The unpolarized regions are concentrated almost entirely on the upper and lower sides of the microholes. According to the series model for composite materials (Equation S11 and Equation S12), the presence of a low-dielectric layer significantly reduces the piezoelectric performance of the composite. The electric field is primarily concentrated in the microhole regions (low dielectric constant). As a result, the electric field strength in the adjacent piezoelectric phase regions (high dielectric constant) along the field direction is insufficient to reach the coercive field, leading to the inability to polarize the upper and lower regions of the microhole structure.

In addition, we compared the polarization response of the (1-0)-3 structure with polar microholes under the same applied polarization field. Due to the significant scale difference between individual CMWCNT and the single crystal, we simulated the contribution of CMWCNTs by setting up macroscopic equivalent floating electrodes and modeled the piezoelectret effect by specifying local charge densities. The simulation result, shown in Fig. S4c, indicates that the volume fraction of the single-crystal region that can be fully polarized increases to 85.34%. By comparing the contour maps of the single-crystal regions in Fig. S4b, c, it is evident that the electric field intensity near the polar microholes is significantly enhanced, thereby enabling a larger portion of the single crystal to be successfully polarized. Meanwhile, a comparison between Fig. S4d, e reveals that, compared with merely increasing the polarization field strength, polar microholes lead to a more uniform electric field distribution across the entire single-crystal region. Therefore, polar microholes can improve the local electric-field distribution and stabilize the polarization regions, thereby ensuring more stable and reliable polarization. Furthermore, the electric field lines in the composite structure under a polarizing electric field were simulated. Fig. S4d, e respectively illustrate the electric field line distributions of the composite filled solely with epoxy and the composite filled with CMWCNTs/epoxy. Compared with Fig. S4d, a more uniform and intensified electric field line distribution is observed around the microholes in Fig. S4e, suggesting that a greater amount of piezoelectric material undergoes effective polarization and thus contributes to the enhanced overall polarization response. This behavior is attributed to the accumulation of net charges in the CMWCNTs/epoxy-filled microholes under a strong electric field, giving rise to a piezoelectret effect that generates additional electric fields around the microholes, as schematically shown in Fig. S4f. Additional simulation results based on PZT-5H ceramics are presented in Fig. S4f–g, providing a systematic analysis of the influence of microhole shape and orientation on the polarization response of piezoelectric materials.

Furthermore, based on the simulation and experimental results, we quantified the independent contributions of the piezoelectret effect and the piezoelectric effect to the SCPC. Based on the above simulation analysis, we found that by introducing polar microholes, the volume fraction of fully polarized single-crystal regions increased by 11.82%. Therefore, by assigning an effective parameter $\overline{d_{33}^{sc}}$ to the piezoelectric phase based on the volume fraction that can be successfully polarized, the effective piezoelectric coefficient $\overline{d_{33}}$ of the SCPC can be predicted. Based on the simulation results, the effective polarized volume fractions (relative to the total pillar volume) of SCPC-2 and SCPC-3 are 62.39% and 69.24%, respectively. And the $\overline{d_{33}^{sc}}$ values of SCPC-2 and SCPC-3 were defined as 837.9 pC/N and 929.9pC/N, respectively. Furthermore, simulation results show that the $\overline{d_{33}}$ values of SCPC-2 and SCPC-3 are 508.6 pC/N and 687.7 pC/N, respectively. Table S3 presents a comparison between the simulation results and the experimental data. It can be seen that the experimental data for SCPC-2 are in close agreement with the simulation results, whereas the experimental values for SCPC-3 are slightly higher than the simulated ones. This is because the $\overline{d_{33}}$ obtained by experiment arises from the synergistic contribution of the piezoelectric effect and the piezoelectret effect, i.e., the DPC mechanism, as shown in equation S13. In contrast, the simulation model only accounts for the contribution from the piezoelectric effect. Therefore, the contribution of the piezoelectret effect to the SCPC can be quantitatively calculated as 38.3 pC/N.

$\overline{d_{33}^{Exp}}=d_{33}^{piezoelectric}+d_{33}^{piezoelectret}$ (S13)

In addition, the mechanical coupling or adhesion quality between the piezoelectric pillars and polymer matrix was also systematic analyzed. The Digital Image Correlation (DIC) system (Aramis-3D 5M, GOM, Germany) was employed to perform non-contact, full-field deformation measurements on the sample surface. Subsequently, standard uniaxial tensile tests were conducted using the multifunctional materials testing machine (Z010, Zwick Roell, Germany). During the experiment, the tensile speed was set to 1 mm/min, and the CCD camera sampling rate was 1 fps. After processing the images using the DIC system's dedicated software, the strain contour maps of the sample at each moment in time can be obtained. Fig. S5b shows interfacial failure of the sample under mechanical tensile loading. The repeated experiments demonstrate that interfacial delamination is the exclusive failure mode occurring at the interface between the piezoelectric single-crystal pillar and the polymer matrix. As shown in the strain field contour maps in Fig. S5c, the critical Von Mises strain at the onset of crack initiation is 2.02% for the sample without CMWCNTs, while it increased to 2.17% for the sample doped with 0.25 wt.% CMWCNTs. The true stress-strain curves of all samples are shown in Fig. S5d. It is clearly observed that when CMWCNTs are doped into the epoxy resin, the interface can withstand higher tensile stress, with the tensile strength increasing as the CMWCNT content rises. For the sample with a 1 wt.% doping level, the true stress at crack initiation is 46.4 MPa, while for the undoped sample, it is 38.8 MPa. This result is consistent with the phenomena observed in the experiments. This enhancement in mechanical performance is primarily attributed to the following factors: (i) Compared to the epoxy resin, CMWCNTs possess a much higher intrinsic stiffness and form a rigid supporting network within the matrix. Under applied load, the epoxy–nanotube network generates multiple friction and pinning effects, preventing interfacial slippage and thereby enhancing the local shear resistance of the surrounding epoxy [S6]. (ii) The MWCNTs used in this work are functionalized with carboxyl groups, allowing them to crosslink with the epoxy resin or form weak chemical bonds (such as hydrogen bonds or polar adsorption) with the ceramic surfaces, further improving interfacial adhesion [S7]. This approach enhances the interfacial reliability of the (1-0)-3 piezocomposite during prolonged working.

**S4 Relationship Between the Properties of (1-0)-3 Piezocomposites and the Structural Parameters**

For 1-3 piezocomposites, the volume fraction of the piezoelectric phase has a significant impact on the overall performance. The critical parameters of piezocomposites with different volume fractions of single-crystal were calculated firstly. Fig. S6 compares the variations of various parameters with single-crystal volume fraction between conventional 1-3 composites and (1-0)-3 composites. As shown in Fig. S6a, the introduction of an ordered microhole structure within the single-crystal pillars significantly reduces the effective dielectric constant, while the decrease in the *d*_33_ is relatively small. Therefore, as shown in Fig. S6c, the *d*_h_*g*_h_ of the (1-0)-3 composite is significantly enhanced. This is beneficial for enhancing the receiving sensitivity of the transducer. For PUTs, a larger *k*_t_ value leads to a wider operating bandwidth and higher electromechanical conversion efficiency. Although the *k*_t_ value of the (1-0)-3 composite decreases slightly, it still exceeds the typical *k*_t_ value (~60%) of conventional 1-3 piezoelectric ceramic composites [S8, S9].

**S5** **Fabrication of the Three SCPCs**

Three types of SCPCs are fabricated based on [011]-oriented relaxor PIN-PMN-PT crystal via a modified method combining 3D printing-assisted dice-and-insert with dice-and-fill techniques, as shown in Fig. 2a. For single-crystal materials, the presence of microcracks can lead to significant performance deterioration. Therefore, preventing the formation and propagation of potential microcracks is essential to maintaining the integrity of the device. First, all single-crystal plates were annealed to release residual stress, and the annealing conditions are shown in Fig. S7b. The fabricated microhole structure is shown in Fig. S7c, demonstrating the well-defined microhole edges and the absence of any visible microcracks in the single-crystal plate. In preliminary work, we found that even under identical processing parameters, cracks are prone to form at the edges of microholes when the drill bit becomes worn, as shown in Fig. S7d. Although it is currently not possible to completely avoid crack formation in every single-crystal sample during machining, undesirable specimens can be eliminated through subsequent screening processes. On the other hand, it is worth noting that, aside from potential cracks introduced during the initial mechanical processing, experiments have shown that when the composite material is exposed to prolonged high-temperature conditions (above 80°C), observable cracks tend to form around the circular holes, as shown in Fig. S7e. This phenomenon is attributed to the mismatch in thermal expansion coefficients between the resin and the single-crystal material, which leads to deformation-induced stress concentration at the interface, ultimately resulting in microcrack formation. However, the actual operating temperature range of the device is between 0°C and 30°C (corresponding to typical ocean temperature fluctuations), which eliminates the potential risk of crack formation associated with prolonged exposure to high temperatures.

For the 1-3 SCPC, the fabrication process is generally consistent with that of the 1-3 composite with nonpolar microholes, except for the steps related to the microhole structure. Fig. S7e compares the embedding configurations of the 3D-printed molds used for the fabrication of the 1-3 SCPC with nonpolar microholes and (1-0)-3 SCPC with poled microholes. Fig. S7f shows three different types of SCPCs, the dimensions of the three SCPCs are 17×17×3.8 mm^3^, 14×14×3.82 mm^3^, and 11.8×12.9×3.91 mm^3^, respectively. This result demonstrates that incorporating ordered microholes into the single-crystal pillars reduces device volume without compromising the high volume fraction of the piezoelectric phase. Notably, following the characterization of the 1-3 SCPC with nonpolar microholes, several strategies, including alternating current polarization, increased polarization voltage, elevated temperature, and extended polarization duration, are employed to enhance its polarization response. These efforts failed to improve its piezoelectric properties and instead led to irreversible performance degradation, therefore, the 1-3 SCPC with nonpolar microholes is not further fabricated into an ultrasonic transducer.

Additionally, finite element simulations were conducted to investigate the stress distribution in the (1-0)-3 structure under ultrasonic excitation at 350 kHz, 400 kHz, and 450kHz. The ultrasonic wave propagates along the thickness direction of the single-crystal pillars. The resulting stress distribution contours are presented in Fig. S7h-j. It can be observed that the introduced microhole structures do not induce additional stress concentration. The regions of high stress generally correspond to the wave peaks of the ultrasonic waves, and therefore, these high-stress regions shift along with the propagation of the waves.

**S6** **Measurement of Longitudinal Wave Velocity in SCPCs**

The piezoelectric composite was placed between the transmitting and receiving transducers, and the sound velocity is calculated using the pulse method. The schematic diagram of the setup used to measure the longitudinal wave velocity in the piezocomposites, along with its optical photograph, is presented in Fig. S9a, b. A fixed distance of 20 mm is maintained between the two ultrasonic transducers. A sinusoidal pulse signal with three cycles is inputted to the transmitting transducer at 500 kHz. The receiving transducer senses the ultrasonic wave and converts it into a voltage signal.

The measuring process can be divided into two stages: (i) The sound speed in water *v_w_* can be calculated by the distance *d* between the two transducers and the time interval *T* between the two pulses*.* (ii) The longitudinal wave sound speed *v_c_* can be calculated by using the second formula in Fig. S9a, where the *t* is the thickness of the piezocomposites. Fig. S9c**-**f are the measured pulse waveform for water, 1-3 SCPC, (1-0)-3 SCPC with nonpolar microholes, and (1-0)-3 SCPC, respectively. The longitudinal wave sound speeds of each single-crystal piezocomposite are calculated to be 3954, 3536, 3707 m/s, respectively.

**S7 Design and Fabrication of the Acoustic Matching and Backing Layers**

**Acoustic matching layers:** To enhance the energy transmission efficiency at the radiating surface interface of the ultrasonic transducer, the design of acoustic impedance matching is essential. It is well known that the acoustic impedance of water is approximately 1.5 MRayls, which is significantly lower than that of SCPCs. Clearly, without proper acoustic impedance matching, a significant portion of the transmitted or received acoustic energy will be lost, leading to degraded performance such as reduced sound transmission efficiency, diminished receiving sensitivity, and a narrow bandwidth. The matching layer or layers provide the necessary acoustic impedance gradient, allowing the ultrasonic transducer to efficiently transmit acoustic energy into the water medium by minimizing acoustic reflections at the interface.

In this work, the ultrasonic transducers are designed with two front matching layers to facilitate acoustic wave transmission. The acoustic impedances *Z*_m1_ (inner) and *Z*_m2_ (outer), as well as their respective thicknesses *t*_m1_ and *t*_m2_, can be estimated using the Krimholtz–Leedom–Mattaei (KLM) model, according to the following equations:

$Z_{m1}=\left(Z_{p}^{4}Z_{l}^{3}\right)^{\frac{1}{7}}$ (S13)

$Z_{m2}=\left(Z_{p}Z_{l}^{6}\right)^{\frac{1}{7}}$ (S14)

$t_{m1}=\frac{\lambda_{m1}}{4}$ (S15)

$t_{m2}=\frac{\lambda_{m2}}{4}$ (S16)

where *Z_p_* and *Z_l_* are the acoustic impedances of the piezoelectric material and load medium (water), respectively. *λ*_m1_ and *λ*_m2_ are the wavelengths of the acoustic wave at the center frequency in the first (inner) and second (outer) matching layers, respectively. For the piezoelectric ultrasound transducers (PUTs) fabricated based on (1-0)-3 SCPC and 1-3 SCPC, the calculated *Z*_m1_ are 6.44, 5.63 MRayls, respectively, and *Z*_m2_ are 2.16, 2.09 MRayls, respectively, as determined using the above formulas. The key parameters corresponding to these layers are summarized in Table S4.

As to the 1^st^ matching layer preparation, the Zirconia oxide powder was selected as the filler and the EPO-TEK 301 epoxy resin was used as the matrix. The ZrO₂ powder and epoxy resin were first mixed at an appropriate weight ratio to form a homogeneous mixture, which was then poured into a mold, centrifuged at 1000 rpm for 12 minutes under vacuum condition, and subsequently cured at room temperature for 24 hours. After polishing the 1^st^ matching layer to the desired thickness, the EPO-TEK 301 epoxy resin was cast onto its surface, degassed under vacuum, and then cured at room temperature for 24 hours. Finally, the transparent epoxy resin layer was polished into the designed thickness as the 2^nd^ matching layer.

**Backing layers:** The backing layer is used to prevent the reflection of incident sound waves return to the transducer, thereby improving signal quality and enhancing imaging clarity. To balance the improvement in bandwidth and the preservation of signal amplitude, a double-layer backing structure is implemented in the ultrasonic transducer.

For the preparation of the 1^st^ backing layer, tungsten (W) powder was used as the filler, and EPO-TEK 301 epoxy served as the matrix. The tungsten (W) powder and epoxy resin were first mixed in an appropriate weight ratio to form a homogeneous mixture, which was then poured into the mold, centrifuged at 1000 rpm for 12 minutes under vacuum condition, and cured at room temperature for 24 hours. After polishing the 1^st^ backing layer to the desired thickness, the 2^nd^ backing layer was prepared on its surface using the same method and then polished to the appropriate thickness after curing. Due to their extremely low density, the hollow glass microspheres effectively reduce the acoustic impedance of the backing material, resulting in smoother impedance matching. Additionally, the hollow glass microspheres can disperse and scatter the sound waves, effectively absorbing them by altering the sound wave propagation path and reducing energy reflection, enhancing the acoustic absorption capacity of the backing layer.

## S8 Insertion Loss and Signal-to-Noise Ratio of PUTs

To evaluate the self-transmit-receive performance of the transducer, the insertion loss (*IL*) is introduced and calculated according to the following equation:

$IL=20\log\frac{V_{out}}{V_{in}}+1.9dB+2.2\times10^{-4}\times2d\times f^{2}$ (S17)

where *V_in_* ​and *V_out_* ​represent the peak-to-peak values of the input sine voltage signal with 20 cycles and the received output voltage of the transducer, respectively. Here, *f* is the excitation frequency, and *d* is the distance between the transducer and the quartz reflector. A fixed value of 1.9 dB is applied to compensate for the reflection loss introduced by the quartz target, while an additional term 2.2×10^-4^×2*d*×*f* ^2^ is used to account for the acoustic attenuation in water.

The signal-to-noise ratio (SNR) test were conducted to evaluate application-relevant metrics. The SNR testing procedure follows the same protocol as the pulse-echo testing of the device, with the only variations being the input voltage or the testing distance. Fig. S12a shows the correlation between SNR and the driving voltage. At lower input voltages, the ultrasonic signal generated by the transducer is relatively weak, while the influence of noise becomes significant. As a result, the SNR is lower at low voltages. At low input voltage levels, a slight increase in the input voltage can result in a significant improvement in the SNR, as the signal becomes much clearer and the relative influence of noise is reduced. When the input voltage is increased from 2 V_p-p_ to 6 V_p-p_, the device's SNR increases from 32.85 to 49.86 dB. As the input voltage increases, the intensity of the ultrasonic signal generated by the piezoelectric transducer increases, leading to an improvement in the SNR. On the other hand, as the propagation distance increases, the intensity of the ultrasonic signal weakens, while the relative strength of the noise remains largely unchanged. Therefore, as shown in Fig. S12b, the SNR decreases with increasing distance. Additionally, as the ultrasonic waves propagate through water, the attenuation rate increases exponentially with the testing distance. Consequently, the SNR of the device decreases more rapidly as the distance increases.

## S9 Long-term Stability and Fatigue Resistance of SCPCs

In addition to the stability and fatigue tests, we have conducted an extensive literature survey on the long-term reliability. By testing the piezoelectric and dielectric properties of the SCPC—including both the 1–3 and 2–2 types—under different hydrostatic pressure conditions (simulating various water depths), the piezoelectric performance of the 1–3 SCPC and 2–2 SCPC remained unchanged when subjected to maximum hydrostatic pressures of 100 MPa (equivalent to a water depth of 10,000 m) and 50 MPa (equivalent to a water depth of 5,000 m), respectively [S10-S11]. On the other hand, although single-crystal materials generally exhibit superior fatigue resistance and field stability compared to polycrystalline ceramics, they may still undergo aging, fatigue, or depolarization under prolonged electric-field loading—particularly when subjected to large-amplitude, alternating, or high-DC-bias fields, and when accompanied by elevated temperatures or unfavorable electrode conditions [S12-S14]. Such degradation is, to some extent, inevitable; nevertheless, it can be substantially mitigated through appropriate doping strategies, electrode optimization, and process improvements.

Based on the aforementioned reports and the long-term active detection tests conducted on the device itself, which inherently requires both acoustic emission and reception, we believe it is reasonable to infer that the proposed SCPC maintains stable piezoelectric performance under the device’s operational conditions and over extended periods of working.

**Supplementary Figures and Tables**

**
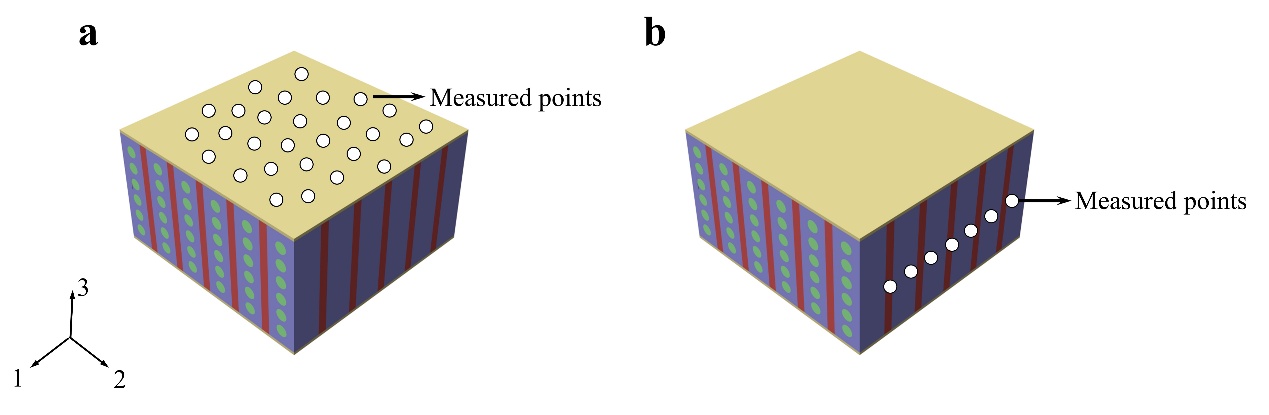
**

**Fig. S1** Schematic diagram of piezoelectric charge coefficient measurement of SCPCs. **a** *d*_33_. **b** *d*_32_/*d*_31_


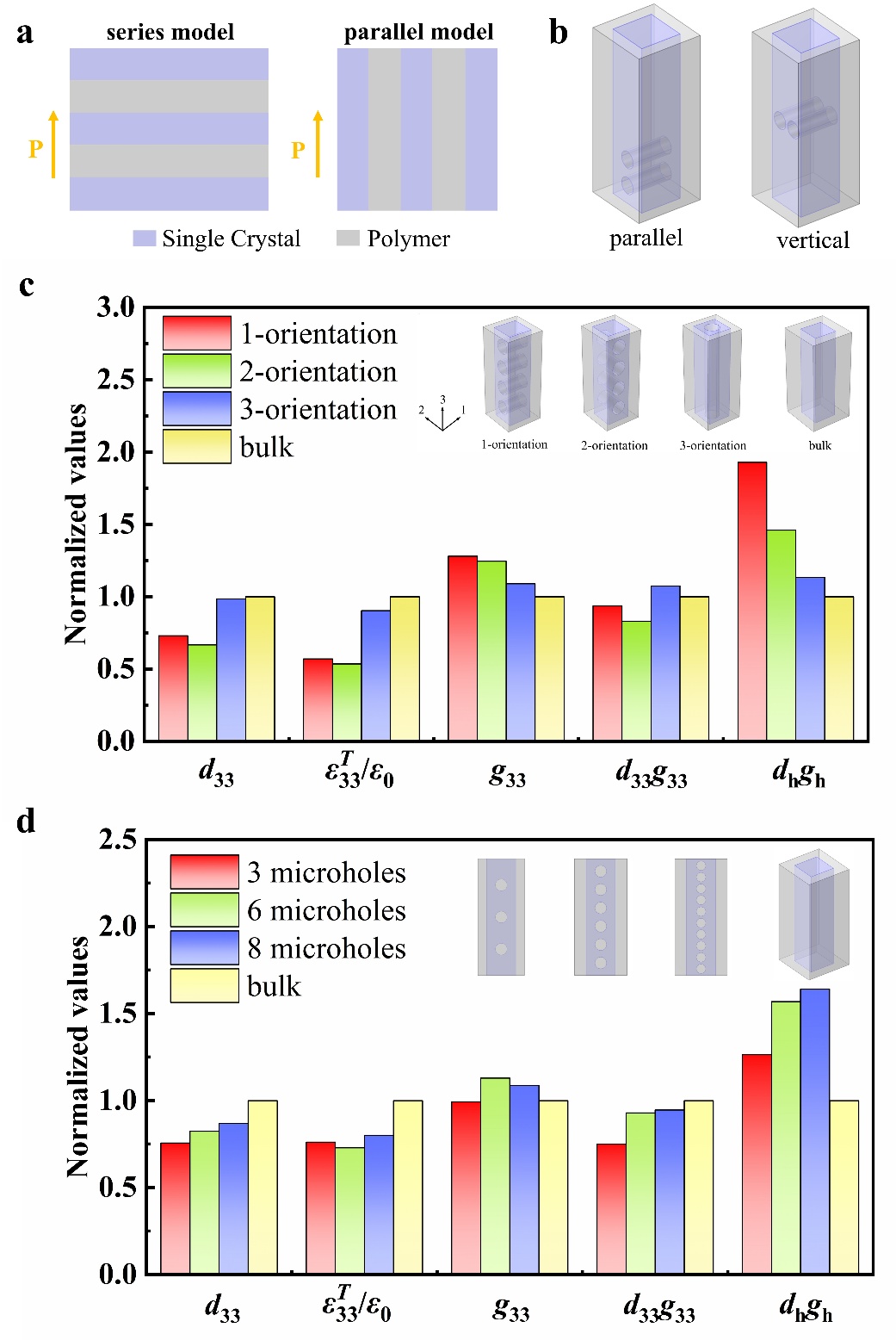


**Fig. S2** The effect of microhole structure on the properties of SCPCs. **a** The series and parallel models for piezoelectric composites. **b** Two typical microhole arrangement configurations. **c** Comparison of normalized values between the composite with different microhole orientations and the microhole-free structure at the same volume fraction. **d** Comparison of normalized values between the composite with different microhole quantities and the microhole-free structure at the same volume fraction

**
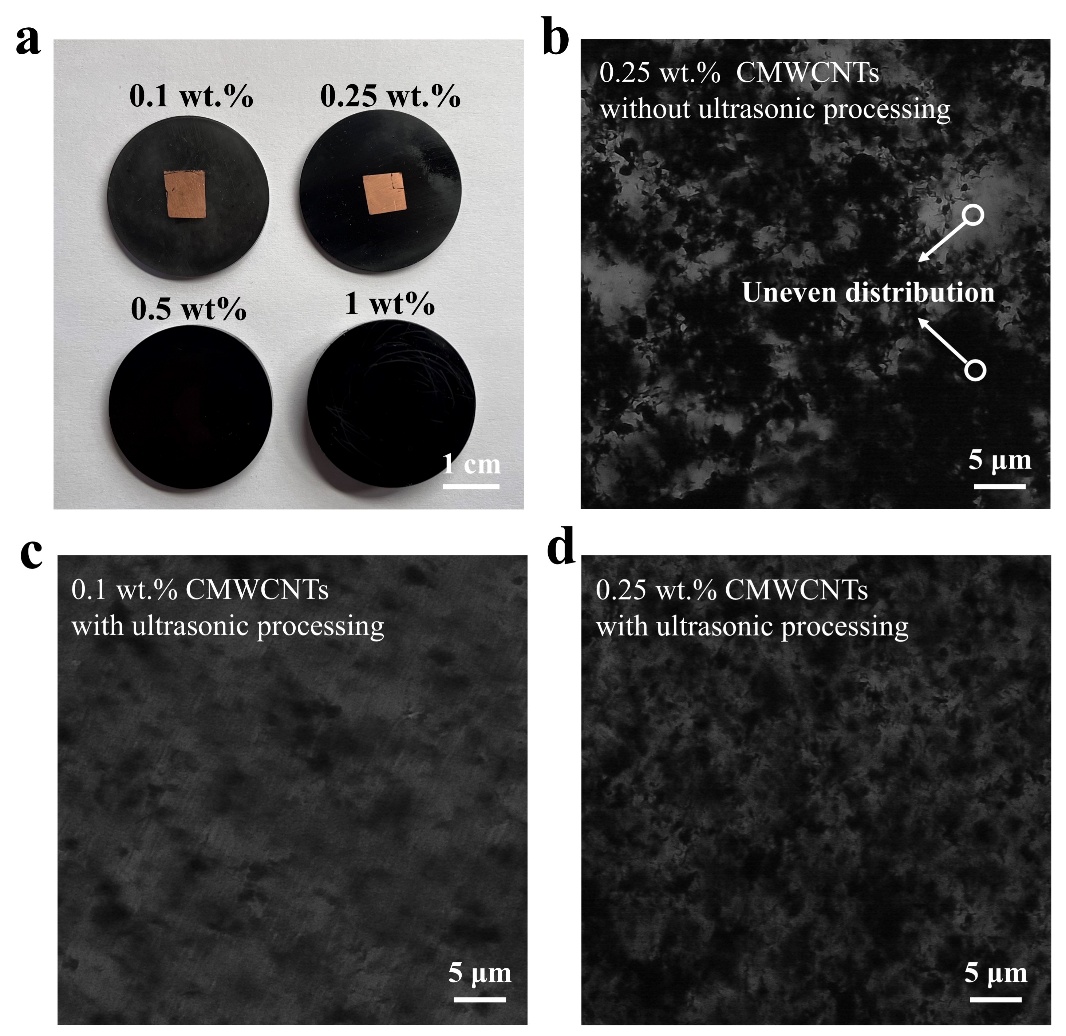
**

**Fig. S3** Optical Microscopy Images of CMWCNTs/epoxy composites. **a** CMWCNTs/epoxy composites with different mass ratios. **b** CMWCNTs/epoxy composites without ultrasonic processing. **c, d** CMWCNTs/epoxy composites with ultrasonic processing


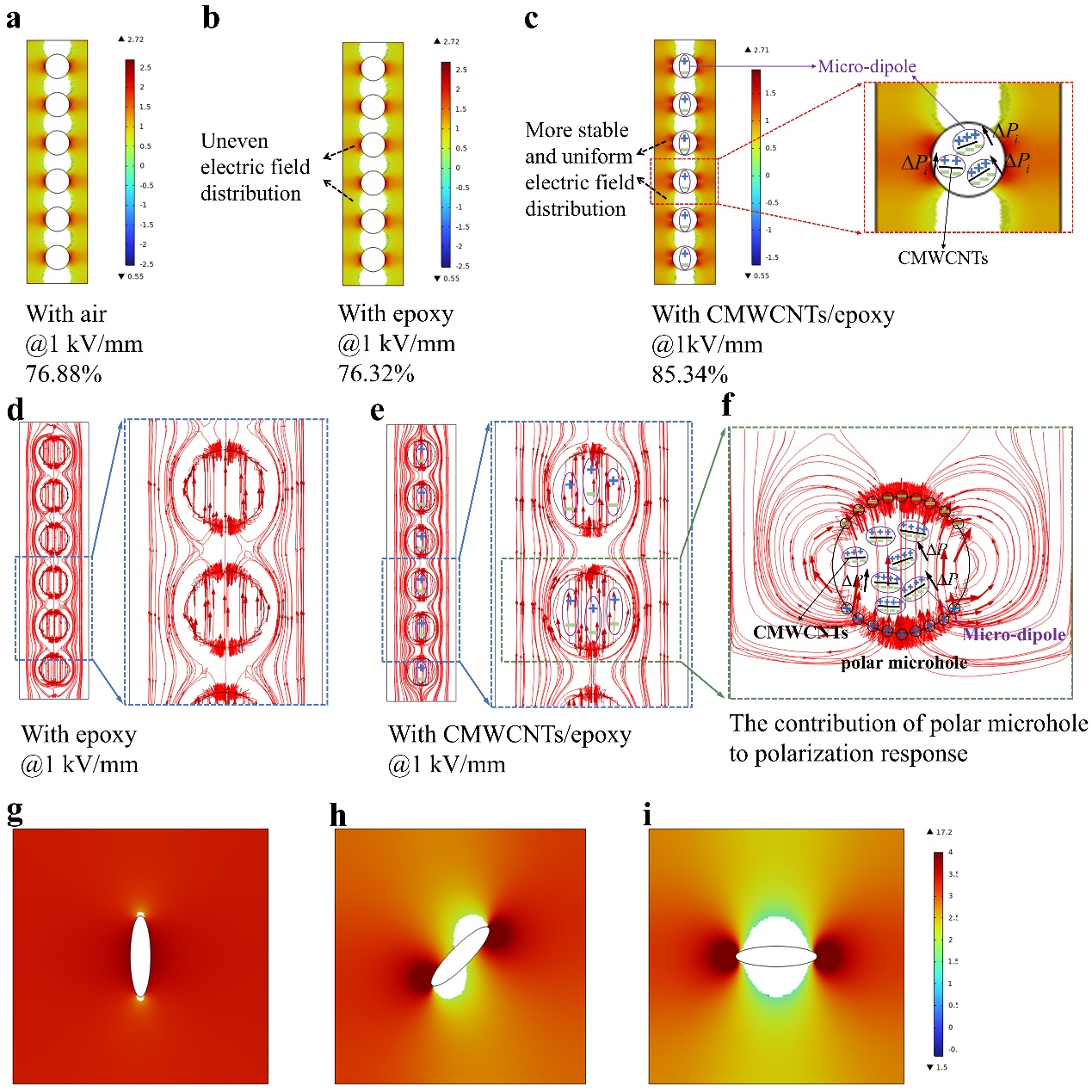


**Fig. S4** Polarization degree analysis of the single-crystal pillars under the applied polarization field**. a** Effect of air-filled microholes on the polarization response. **b** Effect of epoxy-filled microholes on the polarization response. **c** Contribution of polar microholes to the polarization response and electric field distribution. **d** Electric field line diagram of the composite structure with epoxy-filled microholes. **e** Electric field line diagram of the composite structure with polar microholes. **f** The contribution of polar microholes to polarization response. **g–i** Effect of microhole orientation on the polarization response.


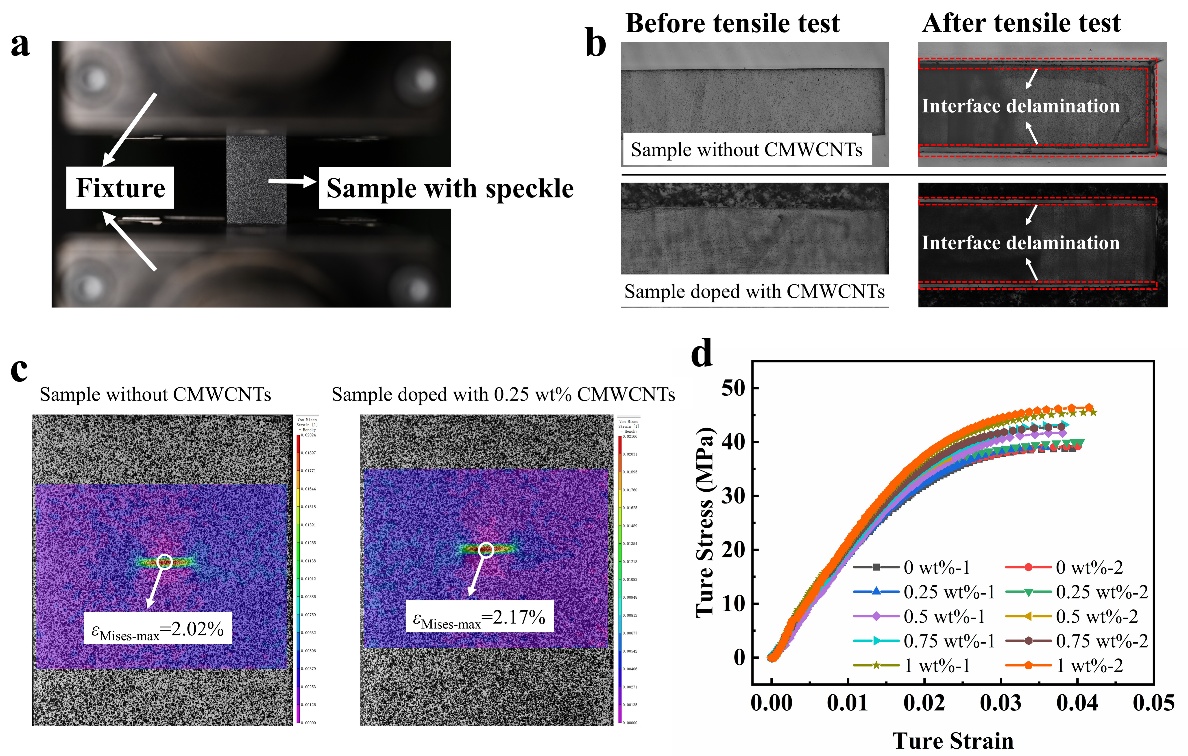


**Fig. S5** Evaluation of interfacial mechanical properties between single-crystal pillars and epoxy resin. **a** Photographs of the sample in the clamped state. **b** Comparison of interface conditions in various samples prior to and following tensile tests. **c** The critical strain field contour map of samples before crack initiation. **d** The true stress-strain curves of all samples


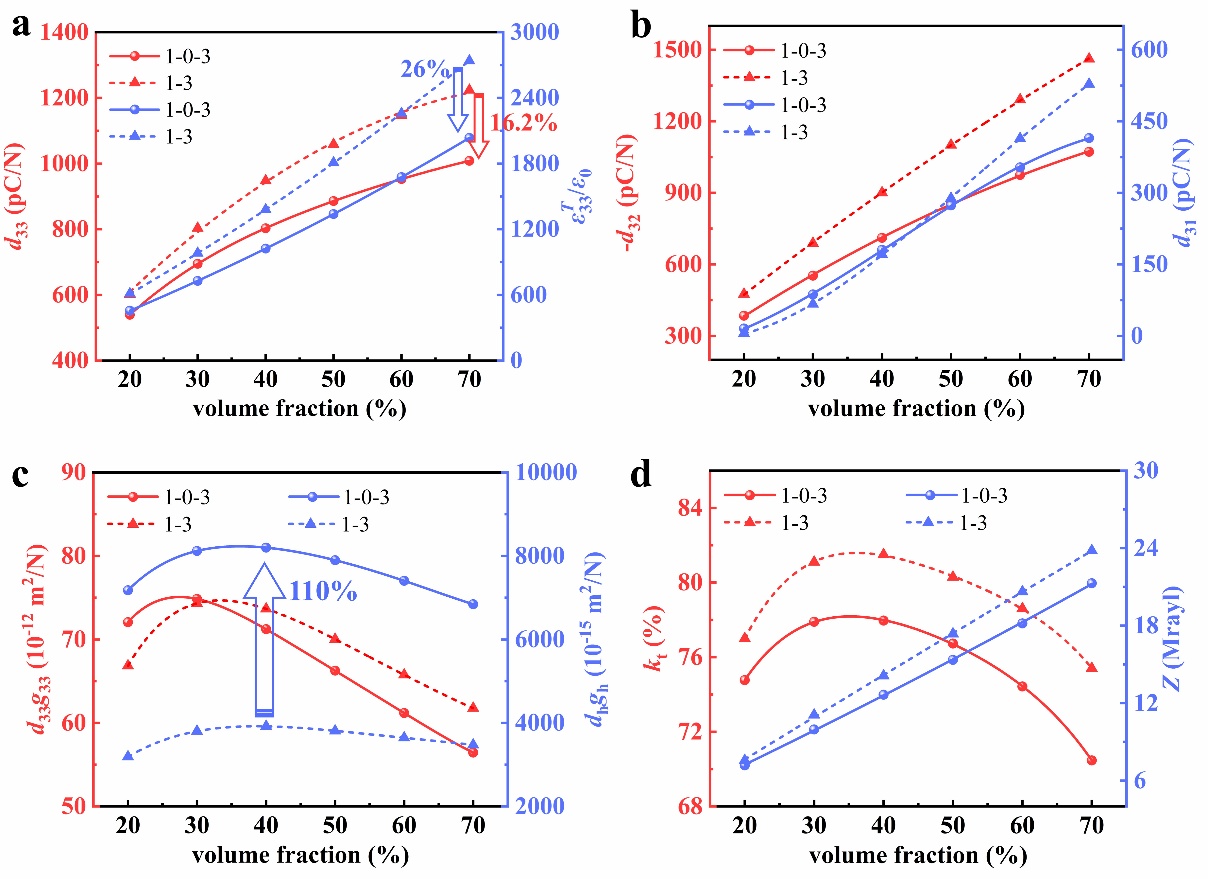


**Fig. S6** The effect of volume fraction on the performance of SCPCs. **a** *d*_33_ and relative dielectric permittivity. **b** *d*_32_ and *d*_31_. **c** Figure of merit *d*_33_*g*_33_ and *d*_h_*g*_h_. (D) *k*_t_ and *Z*


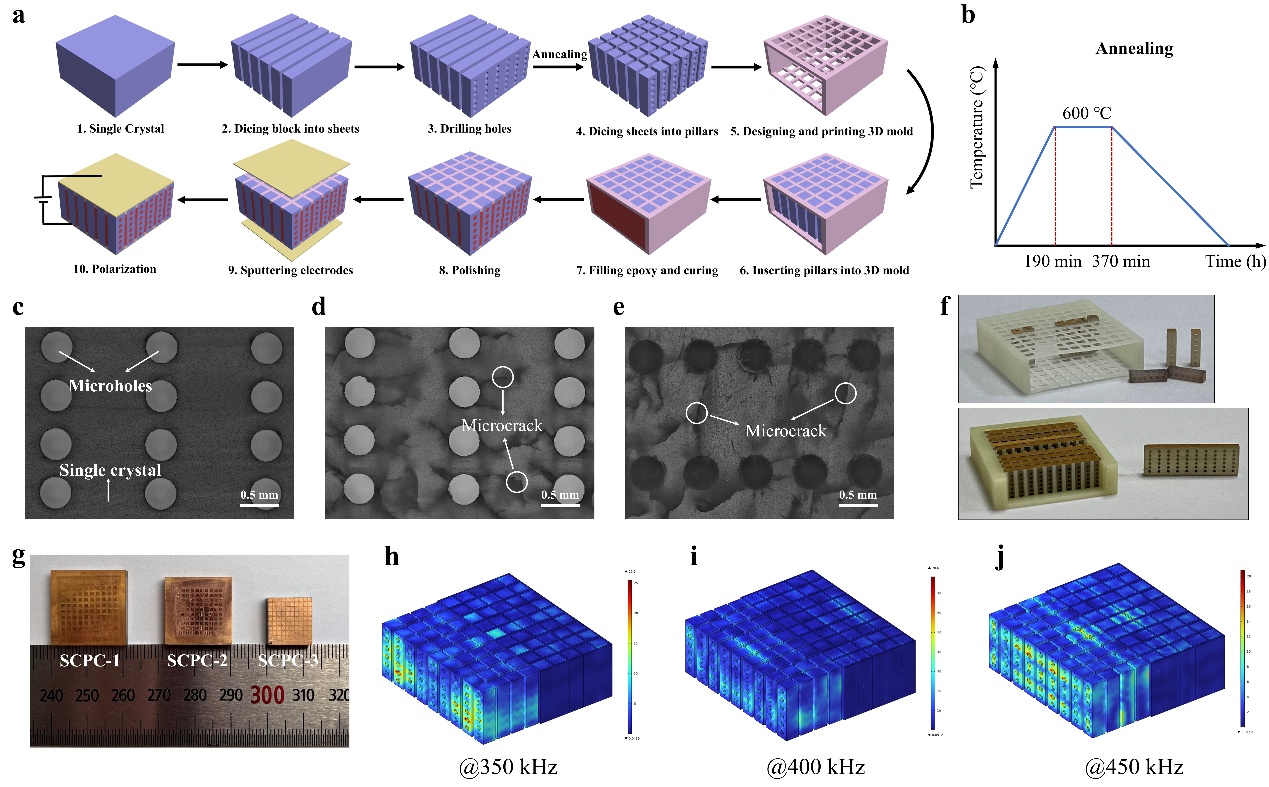


**Fig. S7** Fabrication of SCPCs. **a** Diagram of fabrication processes of 1-3 SCPC with nonpolar microholes. **b** Annealing parameters using a muffle furnace. **c** Microhole structure without microcracks fabricated by precision mechanical processing. **d** Microcracks generated due to drill bit wear during the machining process. **e** Microcracks generated under high-temperature conditions due to the mismatch in the coefficient of thermal expansion of the materials. **f** The embedding configurations of the 3D-printed molds used for the fabrication of the 1-3 SCPC with nonpolar microholes and (1-0)-3 SCPC. **g** Optical image of three different types of SCPCs. **h-j** Stress distribution contours of the (1-0)-3 SCPC under ultrasonic excitation at different frequencies.


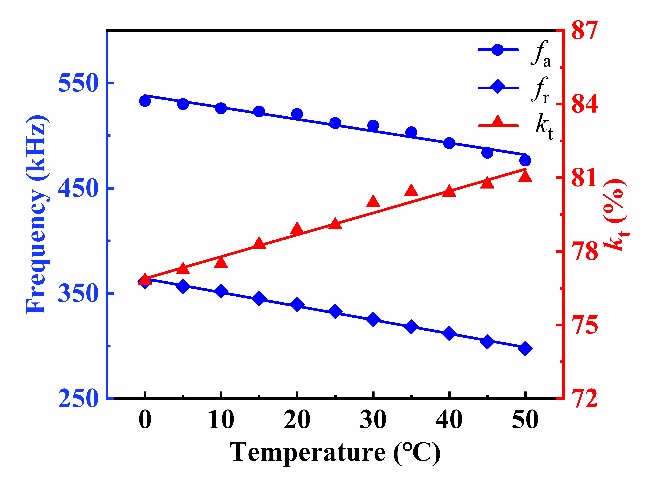


**Fig. S8** The temperature dependence of *f*_r_, *f*_a_ and *k*_t_ of the 1-3 SCPC

**
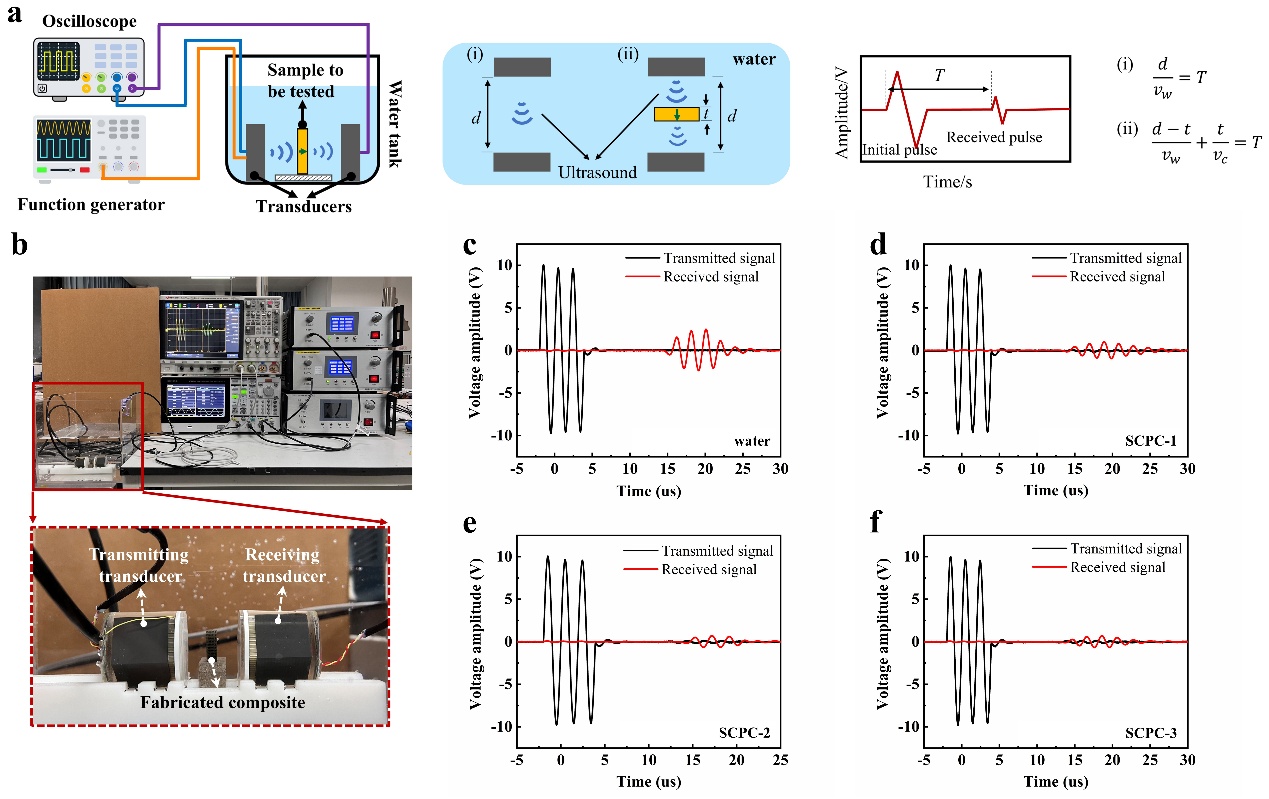
**

**Fig. S9** Measurement of longitudinal wave velocity in SCPCs. **a, b** Setup for measuring longitudinal wave velocity in the piezocomposite: schematic and optical photo. **c, d** The measured pulse waveforms for water, 1-3 SCPC, 1-3 SCPC with nonpolar microholes, and (1-0)-3 SCPC, respectively


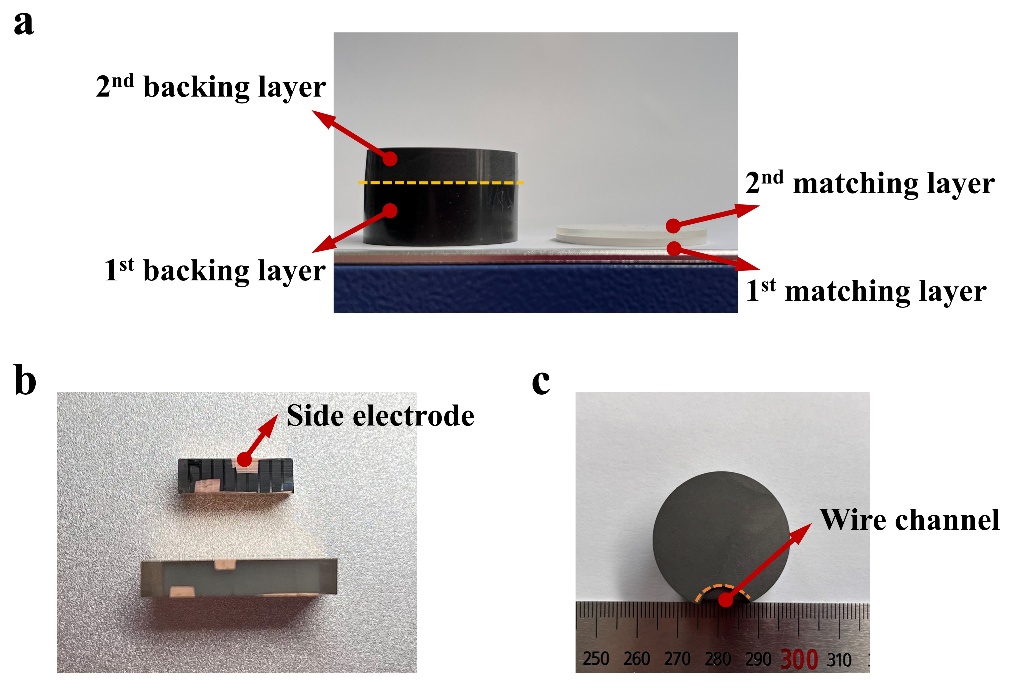


**Fig. S10** Design and fabrication of the acoustic matching and backing layer. **a** The optical photo of the fabricated matching layers and backing layers. **b** Side electrodes are applied to the SCPCs to enable convenient electrical connection via lead wires. **c** Grooved channel for lead wires is ground along the edge of the backing layers


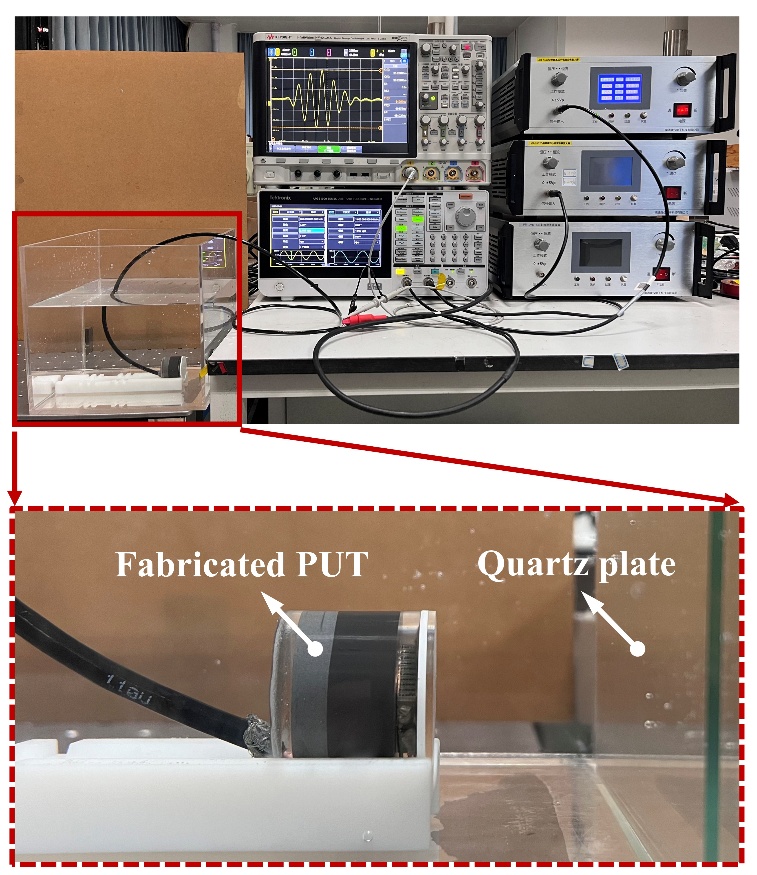


**Fig. S11** The measurement setup of the PUTs for pulse-echo testing


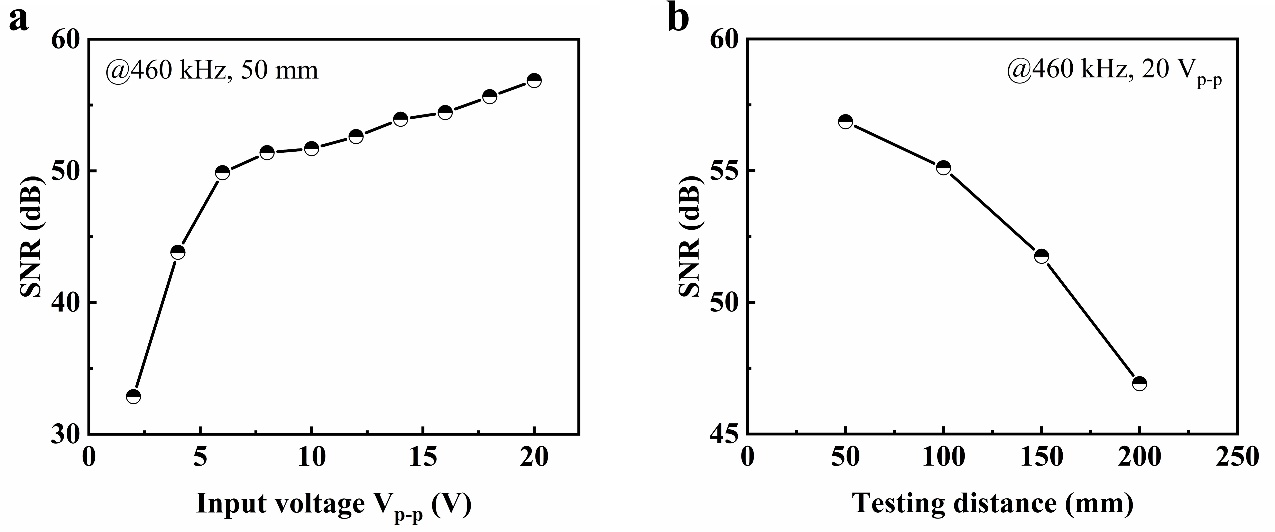


**Fig. R12** SNR test curves of PUT-1: **a** at various input voltages; **b** at different testing distances

**Table S1** The raw data of the d3j (j=1, 2, 3) for the three SCPC samples

| Samples |  | *d*_3j_ (j=1, 2, 3)  (pC/N) | mean ± standard deviation |
| --- | --- | --- | --- |
| 1-3 SCPC | *d*_31_ | 148, 166, 152, 156, 164, 146, 160 | 156 ± 7.1 |
|  | *d*_32_ | -910, -846, -824, -900, -861, -874, -875 | -870 ± 29.9 |
|  | *d*_33_ | 860, 913, 904, 936, 980, 875, 980, 880,  871, 920, 923, 896, 918, 953, 906 | 914.3 ± 35.4 |
| (1-0)-3 SCPC  with nonpolar microholes | *d*_31_ | 108, 85, 92, 105, 90, 106, 86 | 96 ± 9.8 |
|  | *d*_32_ | -449, -433, -456, -424, -416, -419, -455 | -436 ± 15.9 |
|  | *d*_33_ | 551, 455, 464, 529, 485, 545, 490, 475,  471, 520, 523, 506, 489, 500, 487 | 499 ± 29.1 |
| (1-0)-3 SCPC | *d*_31_ | 109, 111, 105, 95, 101, 90, 106 | 102.4 ± 7.6 |
|  | *d*_32_ | -571, -586, -562, -556, -568, -565, -561 | -567 ± 9.7 |
|  | *d*_33_ | 712, 718, 751, 720, 719, 761, 743, 698,  705, 727, 735, 708, 724, 740, 729 | 726 ± 17.6 |

**Table S2** Material parameters of epoxy resin

| $\rho$ (kg/m^3^) | $\varepsilon/\varepsilon_{0}$ | *E* (GPa) | Poisson's ratio |
| --- | --- | --- | --- |
| 1150 | 4 | 3 | 0.35 |

**Table S3** Comparison of the simulated and experimental effective piezoelectric coefficients

|  | SCPC-2 | |  | SCPC-3 | |
| --- | --- | --- | --- | --- | --- |
| $\overline{d_{33}}$ (pC/N) | Experimental | Simulated |  | Experimental | Simulated |
|  | 499 | 508.6 |  | 726 | 687.7 |

**Table S4** The parameters of the acoustic material used for the PUTs fabrication

|  | Materials | Function | Density  (kg/m^3^) | Sound velocity  (m/s) | Thickness  (mm) | Acoustic impedance  (MRayls) |
| --- | --- | --- | --- | --- | --- | --- |
| (1-0)-3 SCPC based PUT | Epoxy 301/ZrO_2_ (1:2.2) | 1^st^ ML | 2444 | 2628 | 1.5 | 6.4 |
|  | Epoxy 301 | 2^nd^ ML | 1150 | 2650 | 1.51 | 3.05 |
|  | Epoxy/Tungsten (1:5.2) | 1^st^ BL | 5173 | 1591 | 11.8 | 8.23 |
|  | Epoxy/Tungsten/ microspheres (3:6:1) | 2^nd^ BL | 1593 | 1327 | 6 | 2.11 |
| 1-3 SCPC based PUT | Epoxy 301/ZrO_2_ (1:1.6) | 1^st^ ML | 2272 | 2495 | 1.6 | 5.67 |
|  | Epoxy 301 | 2^nd^ ML | 1150 | 2650 | 1.62 | 3.05 |
|  | Epoxy/Tungsten (1:4) | 1^st^ BL | 4670 | 1590 | 11.86 | 7.43 |
|  | Epoxy/Tungsten/ microspheres (3:6:1) | 2^nd^ BL | 1593 | 1327 | 6 | 2.11 |

ML: matching layer, BL: backing layer

**Table S5** Comparison of the sensitivity properties of the PUT-1 ultrasonic transducer with those of other reported ultrasound transducers

| Material | Size  (mm^3^) | *f*_c_  (Hz) | RVS_max_  (dB) | BW_-3 dB_  (kHz) | *IL*  (dB) | Refs. |
| --- | --- | --- | --- | --- | --- | --- |
| (1-0)-3 PIN-PMN-PT single-crystal containing polar microholes/epoxy | 11.8×12.9 | 455 k | −184.3 | 130 | −14.4 | **This work** |
| 1-3 PNN-PZT/epoxy | 15×15 | 350 k | −206 | 104 | - | [25] |
| 1-3 PZT-5H/epoxy | 5×5 | 3.26 M | - | - | −32.9 | [31] |
| 1-3 porous PZT composites | R5.75 | 120 k | −199 | 26 | - | [61] |
| 1-3 PZT-5A/epoxy | 187×47 | 222 k | −198.8 | 15.4 | - | [65] |
| 1-3 PIN-PMN-PT single-crystal/epoxy | 15×15 | 520 k | −191.8 | 140 | −18.9 | [24] |
| 1-3 PZN-PT single-crystal/epoxy | 3×3 | 20 M | - | - | −23.9 | [30] |
| 1-3 Mn: PIN-PMN-PT single-crystal/epoxy | 5×5 | 3 M | - | - | −25.2 | [31] |

**Supplementary** **References**

1. E. Sun, S. Zhang, J. Luo, T.R. Shrout, W. Cao, Elastic, dielectric, and piezoelectric constants of Pb(In_1/2_Nb_1/2_)O_3_–Pb(Mg_1/3_Nb_2/3_)O_3_–PbTiO_3_ single crystal poled along [011] c. Appl. Phys. Lett. **97**(3), 032902 (2010). <https://doi.org/10.1063/1.3466906>
2. M. Koç, F. Tatardar, N.N. Musayeva, S. Guluzade, A. Sarı et al., The piezoelectric properties of three-phase electrospun PVDF/PZT/Multiwalled Carbon Nanotube composites for energy harvesting applications. J. Alloys Compd. **1003**, 175578 (2024). <https://doi.org/10.1016/j.jallcom.2024.175578>
3. K. Sharma, K. Bhunia, S. Chatterjee, M. Perumalsamy, A.A. Saj et al., Deep learning-assisted organogel pressure sensor for alphabet recognition and bio-mechanical motion monitoring. Nanomicro Lett. **18**(1), 63 (2025). https://doi.org/10.1007/s40820-025-01912-z
4. M. Sun, S. Wang, Y. Liang, C. Wang, Y. Zhang et al., Flexible graphene field-effect transistors and their application in flexible biomedical sensing. Nanomicro Lett. **17**(1), 34 (2024). <https://doi.org/10.1007/s40820-024-01534-x>
5. H. Kim, B. Rigo, G. Wong, Y.J. Lee, W.-H. Yeo, Advances in wireless, batteryless, implantable electronics for real-time, continuous physiological monitoring. Nanomicro Lett. **16**(1), 52 (2023). <https://doi.org/10.1007/s40820-023-01272-6>
6. Y.-K. Choi, K.-I. Sugimoto, S.-M. Song, Y. Gotoh, Y. Ohkoshi et al., Mechanical and physical properties of epoxy composites reinforced by vapor grown carbon nanofibers. Carbon **43**(10), 2199–2208 (2005). <https://doi.org/10.1016/j.carbon.2005.03.036>
7. K.T. Kim, S.I. Cha, S.H. Hong, S.H. Hong, Microstructures and tensile behavior of carbon nanotube reinforced Cu matrix nanocomposites. Mater. Sci. Eng. A **430**(1–2), 27–33 (2006). <https://doi.org/10.1016/j.msea.2006.04.085>
8. S. Hao, C. Zhong, Y. Zhang, Y. Chen, L. Wang et al., Flexible 1-3 piezoelectric composites with soft embedded conductive interconnects for underwater acoustic transducers. ACS Appl. Electron. Mater. **5**(5), 2686–2695 (2023). <https://doi.org/10.1021/acsaelm.3c00155>
9. H. Qin, H. Lu, J. Zhou, Y. Zhang, Effect of thickness on the performance parameters of modified 1–3 piezoelectric composites. Ceram. Int. **49**(7), 10928–10935 (2023). <https://doi.org/10.1016/j.ceramint.2022.11.286>
10. T. Wang, X. Zhao, H. Du, S. Xia, G. Li et al., Large-area piezoelectric single crystal composites *via* 3-D-printing-assisted dice-and-insert technology for hydrophone applications. IEEE Trans. Ultrason. Ferroelectr. Freq. Control **68**(10), 3241–3248 (2021). <https://doi.org/10.1109/TUFFC.2021.3085842>
11. L. Li, S. Zhang, Z. Xu, X. Geng, F. Wen et al., Hydrostatic piezoelectric properties of [011] poled Pb(Mg_1/3_Nb_2/3_)O_3_-PbTiO_3_ single crystals and 2-2 lamellar composites. Appl. Phys. Lett. **104**(3), 032909 (2014). <https://doi.org/10.1063/1.4862984>
12. Y.A. Genenko, J. Glaum, M.J. Hoffmann, K. Albe, Mechanisms of aging and fatigue in ferroelectrics. Mater. Sci. Eng. B **192**, 52–82 (2015). <https://doi.org/10.1016/j.mseb.2014.10.003>
13. J. Glaum, M. Hoffman, Electric fatigue of lead-free piezoelectric materials. J. Am. Ceram. Soc. **97**(3), 665–680 (2014). <https://doi.org/10.1111/jace.12811>
14. F. Fang, W. Yang, F.C. Zhang, H.S. Luo, Fatigue crack growth for BaTiO_3_ ferroelectric single crystals under cyclic electric loading. J. Am. Ceram. Soc. **88**(9), 2491–2497 (2005). <https://doi.org/10.1111/j.1551-2916.2005.00436.x>
